# Angulin-1 (LSR) Affects Paracellular Water Transport, However Only in Tight Epithelial Cells

**DOI:** 10.3390/ijms22157827

**Published:** 2021-07-22

**Authors:** Carlos Ayala-Torres, Susanne M. Krug, Rita Rosenthal, Michael Fromm

**Affiliations:** Clinical Physiology/Nutritional Medicine, Medical Department, Division of Gastroenterology, Infectiology and Rheumatology, Charité—Universitätsmedizin Berlin, 12203 Berlin, Germany; carlos-mario.ayala-torres@charite.de (C.A.-T.); susanne.m.krug@charite.de (S.M.K.); rita.rosenthal@charite.de (R.R.)

**Keywords:** angulin-1, LSR, tricellulin, tricellular tight junction, paracellular water transport, tight epithelium, MDCK C7 cells, intermediate-tight epithelium, HT-29/B6 cells

## Abstract

Water transport in epithelia occurs transcellularly (aquaporins) and paracellularly (claudin-2, claudin-15). Recently, we showed that downregulated tricellulin, a protein of the tricellular tight junction (tTJ, the site where three epithelial cells meet), increased transepithelial water flux. We now check the hypothesis that another tTJ-associated protein, angulin-1 (*alias* lipolysis-stimulated lipoprotein receptor, LSR) is a direct negative actuator of tTJ water permeability depending on the tightness of the epithelium. For this, a tight and an intermediate-tight epithelial cell line, MDCK C7 and HT-29/B6, were stably transfected with CRISPR/Cas9 and single-guide RNA targeting angulin-1 and morphologically and functionally characterized. Water flux induced by an osmotic gradient using 4-kDa dextran caused water flux to increase in angulin-1 KO clones in MDCK C7 cells, but not in HT-29/B6 cells. In addition, we found that water permeability in HT-29/B6 cells was not modified after either angulin-1 knockout or tricellulin knockdown, which may be related to the presence of other pathways, which reduce the impact of the tTJ pathway. In conclusion, modulation of the tTJ by knockout or knockdown of tTJ proteins affects ion and macromolecule permeability in tight and intermediate-tight epithelial cell lines, while the transepithelial water permeability was affected only in tight cell lines.

## 1. Introduction

Adequate transport of solutes and water across epithelial barriers is indispensable for maintaining normal physiological homeostasis in all animals [1,2]. Fluid is moved either across the plasma membranes of the cells that comprise the epithelial layer (transcellular transport) or between these cells (paracellular transport). The discovery of aquaporin water channels provided a first molecular basis for transcellular water movement [3].

Paracellular transport involves specialized structures called tight junctions (TJ) that regulate the flow of solutes through paracellular pathways and maintain cell polarity, thereby functioning as a barrier of epithelial and endothelial cellular sheets [4]. Tricellular tight junctions (tTJs) form at the convergence of bicellular tight junctions (bTJs) where three epithelial cells meet in polarized epithelia [5,6]. Claudin family proteins and occludin are main components of bicellular TJs, which are important for the barrier function and permselectivity [7,8,9,10]. To date, 27 members of the claudin family have been identified in humans. Many of these have sealing functions (claudins 1, 3, 5, 11, 14, 19) while some claudins form channels across TJs, which are permeable either for cations (claudins 2, 10b, 15) or for anions (claudins 10a, 17). For several claudins, their effects on epithelial barriers are inconsistent and the function is unclear (claudins 4, 7, 8, 16) [11]. The claudin composition of the TJ mainly determines the tightness of an epithelium or an epithelial cell line, respectively.

In addition to their role as channels for small cations, Rosenthal et al. demonstrated that claudin-2 and claudin-15 also transport water [12,13,14].

The mechanisms that determine the use of paracellular versus transcellular routes for water transport are multifaceted. For example, in the kidney, transport of water is achieved in part through the paracellular pathway in the proximal nephron [15], whereas in the distal nephron and collecting duct, it takes place through transcellular routes [16]. Mutations in claudins have been identified to cause hereditary diseases, demonstrating that regulation of the paracellular permeability is crucial to the normal functions of various organs [11].

The tricellular TJ is more complex than bTJ. In the center of tTJ, bTJ elements from each of the three cells come together to form a central tube of about 1 μm in length and 10 nm in diameter [17]. To date, two types of integral membrane proteins, tricellulin and angulin family proteins, are identified as molecular components of tTJ [18]. Tricellulin, which was the first tTJ protein to be identified [5], plays a critical role in sealing the tTJ against the passage of solutes of up to 10 kDa [19,20], and it was recently found that it also plays a role in the paracellular water transport in the tight epithelial cell line, MDCK C7 [21].

As said, angulins contribute to forming the central element of the tTJ. Angulin family proteins, comprising lipolysis-stimulated lipoprotein receptor (LSR), immunoglobulin-like domain containing receptor-1 (ILDR1) and -2 (ILDR2), are type-I transmembrane proteins with an extracellular immunoglobulin-like domain. Due to their common structure and function as tTJs-associated membrane proteins, LSR, ILDR1, and ILDR2 were designated as angulin-1, angulin-2, and angulin-3, respectively [22].

The original role of LSR/angulin-1 was described in lipid metabolism studies, as LSR was first discovered to be expressed in hepatocytes, where it plays a role in the clearance of triglyceride-rich lipoproteins and low-density lipoproteins, and also acts as an apolipoprotein B/E–containing lipoprotein receptor [23]. At tricellular contacts, angulin-1 recruits tricellulin, and the interaction between the cytoplasmic domain of angulin-1 and the C-terminal cytoplasmic domain of tricellulin is required for this recruitment [24,25]. Angulin-1 together with tricellulin are required for full barrier function of epithelial cells with high transepithelial electrical resistance [25]. Loss or downregulation of angulin-1 disrupts the barriers with relocalization of tTJ molecule tricellulin in various cell types [24,26]. In addition, loss of angulin-1 enhances cancer cell motility [27,28] and the LSR knockout mice die before embryonic day 15.5 (E15.5), but the cause of death remains unclear [29].

The LSR-related proteins immunoglobulin-like domain containing receptors angulin-2 and angulin-3 are also expressed complementarily in many epithelial cell types at tricellular contacts of many epithelial cells and recruit tricellulin [22], for instance, it is known that angulin-1 and angulin-2 are co-expressed in the large intestine and the kidney [22].

As for the tTJ, Gong et al. provided evidence that water transport is increased in isolated kidney tubules in the absence of angulin-2, but is normally inhibited in the presence of angulin-2 [30]. In contrast to this, Hempstock et al. found in the colon and kidney of angulin-2 KO mice no detectable abnormalities in water transport and maintained barrier function of the epithelia. They concluded that angulin-1 changes its expression pattern and location, which compensates for the loss of angulin-2 [31].

These findings led us to hypothesize that angulin-1 is a direct negative actuator of tTJ water permeability depending on the tightness of the epithelium, in this respect similar to tricellulin. Therefore, we aimed to characterize the effect of angulin-1 expression on water transport in a tight and an intermediate-tight epithelial cell line, MDCK C7 and HT-29/B6, respectively. In order to define the tightness of these cell lines, we previously performed a two-path impedance spectroscopy study where we analyzed paracellular, transcellular, and transepithelial resistance in MDCK C7, HT-29/B6, and MDCK C11 cells. As a result, MDCK C7 cells were defined as a tight and HT-29/B6 cells as a moderate tight epithelial cell line, as defined by the ratio R^para^/R^trans^ and the absolute resistance values of the two pathways [32]. In our present study, we found that the water passage through the tTJ was changed in the absence of angulin-1 only in MDCK C7 cells, thus in a tight epithelium.

## 2. Results

### 2.1. Establishment of Angulin-1 Knockout in MDCK C7 and HT-29/B6 Cells

**Figure 1:**
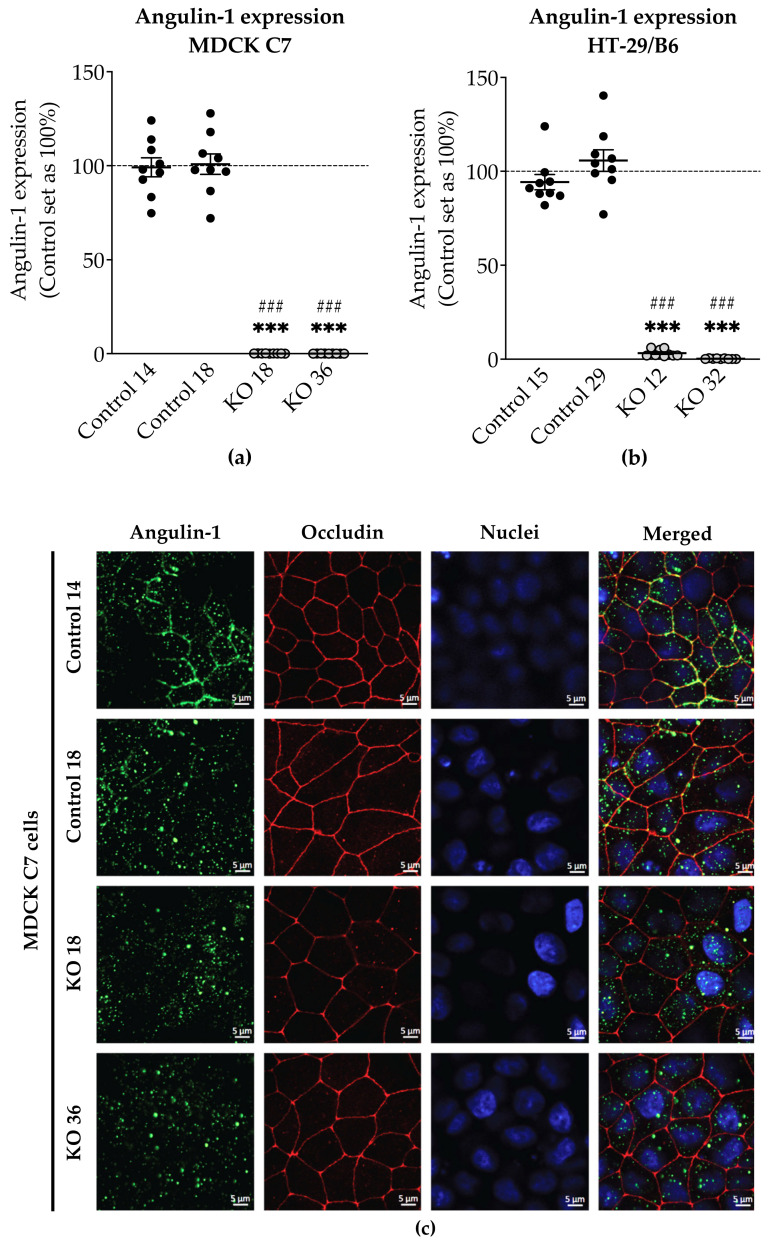

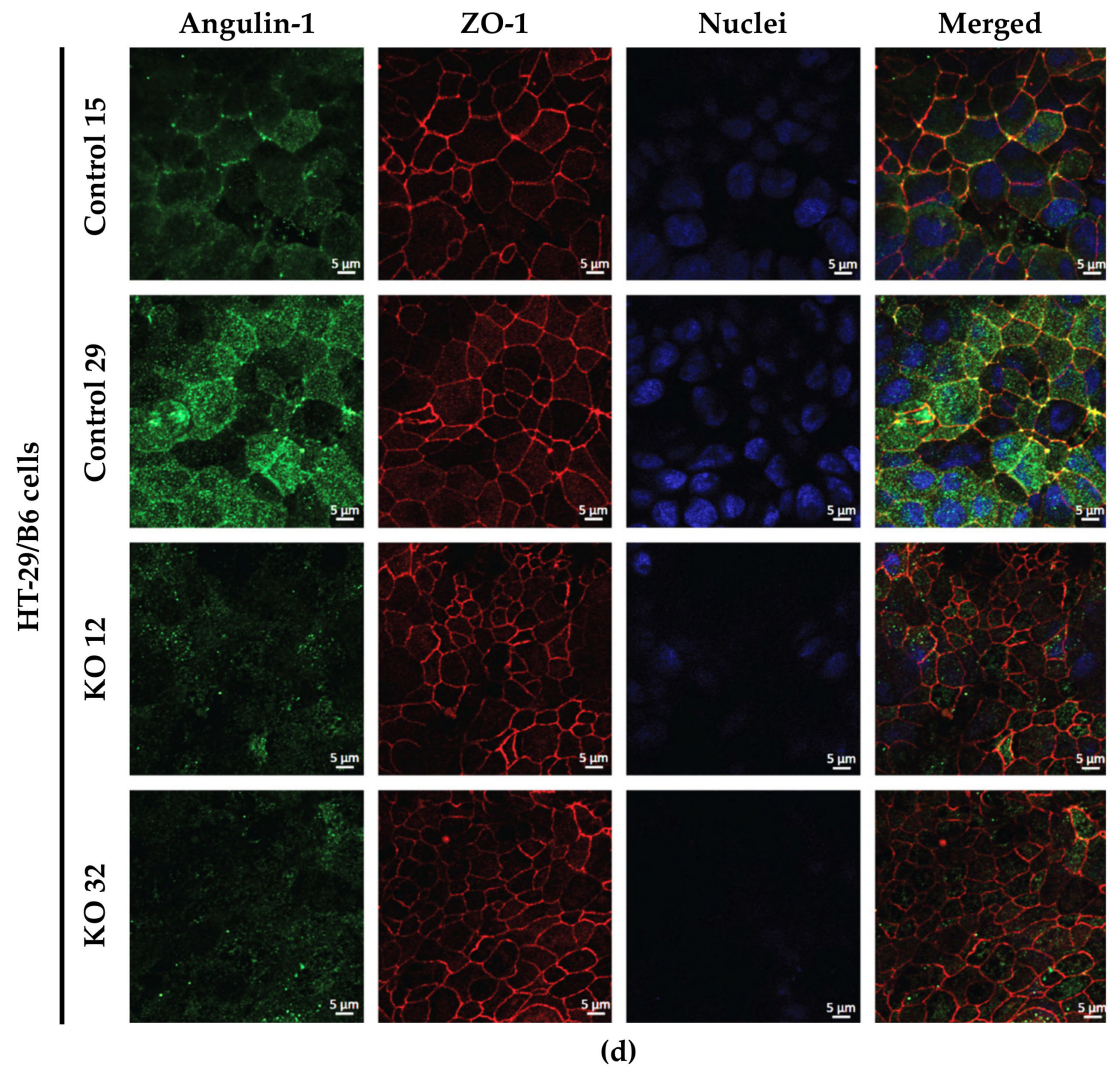
Expression and localization of angulin-1 in MDCK C7 and HT-29/B6 cells. Densitometric analysis of angulin-1 protein expression levels in control and angulin-1 knockout (**a**) MDCK C7 and (**b**) HT-29/B6 cells. sgRNA targeting angulin-1 led to a high decrease in angulin-1 expression in both cell lines (*n* = 9, MDCK C7: *** *p* ≤ 0.001 with regard to control 14 and ^###^ *p* ≤ 0.001 with regard to control 18 and HT-29/B6: *** *p* ≤ 0.001 with regard to control 15 and ^###^ *p* ≤ 0.001 with regard to control 29). Immunostaining of angulin-1 in the clones used throughout this study in (**c**) MDCK C7 and (**d**) HT-29/B6 cells. In the KO clones of both cell lines, angulin-1 disappeared from the tTJ in comparison with their controls. Angulin-1: green; Occludin or ZO-1: red; DAPI (nuclei): blue.

After transfection of MDCK C7 and HT-29/B6 cells with CRISPR/Cas9, HDR plasmids and sgRNA targeting angulin-1, puromycin-resistant cell clones were screened for angulin-1 knockout by Western blotting. In this study, two angulin-1 knockout clones and their controls were investigated in both cell lines (KO 18 and KO 36 and their respective control clones 14 and 18 in MDCK C7 cell line; and KO 12 and KO 32 and their respective controls 15 and 29 in HT-29/B6 cell line). The angulin-1 KO clones in both cell lines were mono-allelic knockouts (Figure 1a,b, Supplementary Tables S1 and S2).

The localization of angulin-1 in the controls and its removal from the TJ in the knockouts was confirmed for all clones as dots at the tTJ by immunofluorescence confocal laser-scanning microscopy. ZO-1 [33] in HT-29/B6 cells and occludin [34] in MDCK C7 cells served as a TJ marker (Figure 1c,d).

### 2.2. Effects of Angulin-1 Knockout on Endogenous Proteins of MDCK C7 and HT-29/B6 Cells

**Figure 2:**
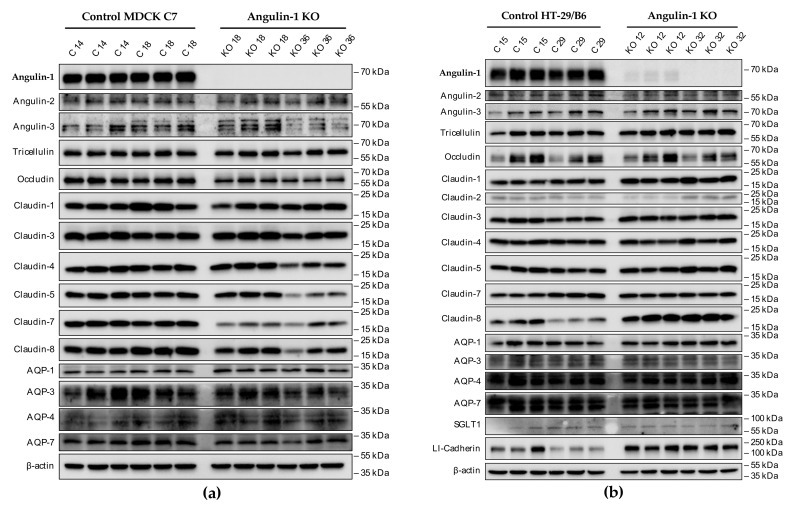

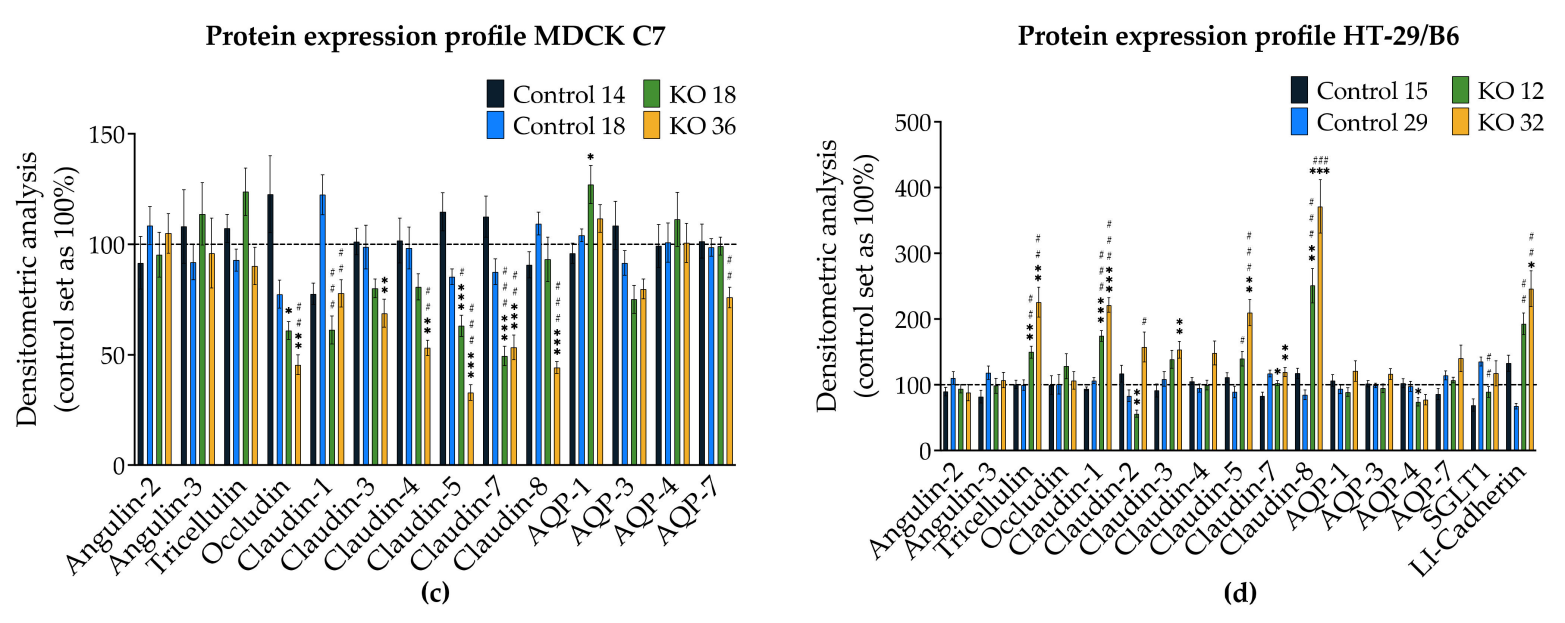
Angulin, tricellulin, occludin, claudin, and aquaporin (AQP) expression in control and angulin-1 knockout cells. Representative Western blots of angulin-1 knockout in (**a**) MDCK C7 and (**b**) HT-29/B6 cells. Densitometric analysis of protein expression levels in (**c**) MDCK C7 and (**d**) HT-29/B6 cells after angulin-1 knockout in comparison to the corresponding vector-transfected controls (*n* = 9, N = 3). β-Actin was used as an internal control for normalization to protein content. Statistical analysis was performed using one-way ANOVA test to compare between the four clones in both cell lines (MDCK C7: * *p* ≤ 0.05, ** *p* ≤ 0.01, *** *p* ≤ 0.001 with regard to control 14 and ^#^ *p* ≤ 0.05, ^##^ *p* ≤ 0.01, ^###^ *p* ≤ 0.001 with regard to control 18 and HT-29/B6: * *p* ≤ 0.05, ** *p* ≤ 0.01, *** *p* ≤ 0.001 with regard to control 15 and ^#^ *p* ≤ 0.05, ^##^ *p* ≤ 0.01, ^###^ *p* ≤ 0.001 with regard to control 29).

Knockout of one TJ protein may cause relevant variation in other proteins potentially involved in transepithelial water transport. Therefore, we examined levels of tricellulin, the three angulins, occludin, several claudins, aquaporin (AQP) water channels, AQP-1, -3, -4 and -7, SGLT1, and LI-cadherin (Figure 2a,b). As known from previous studies, claudin-2 is not expressed in MDCK C7 cells [12,35]. The densitometric analysis revealed some clonal variability in TJ protein expression between the knockout clones and the controls.

In detail, occludin, claudin-1, -3, -4, -5, -7, -8, and AQP-7 were reduced in the MDCK C7 angulin-1 KO 36 clone (Figure 2c). The MDCK C7 angulin-1 KO 18 clone showed a reduction in occludin, claudin-1, -5, and -7 and a slight increase in AQP-1 compared with the controls (Figure 2c). On the other hand, angulin-1 knockout resulted in increased levels of tricellulin, claudin-1, -5, -7 and -8 and LI-cadherin and decreased levels of claudin-2, AQP-4 and SGLT1 in HT-29/B6 angulin-1 KO 12 clone (Figure 2d). The HT-29/B6 angulin-1 KO 32 clone showed an upregulation of tricellulin, claudin-1, -2, -3, -5, -7, -8, and LI-cadherin (Figure 2d).

Clonal variability in protein expression was also observed between both control clones and both KO clones. None of the other two angulins were significantly altered in the knockout clones of both cell lines.

### 2.3. Effects of Angulin-1 Knockout on Tricellulin Localization in MDCK C7 and HT-29/B6 Cells

**Figure 3:**
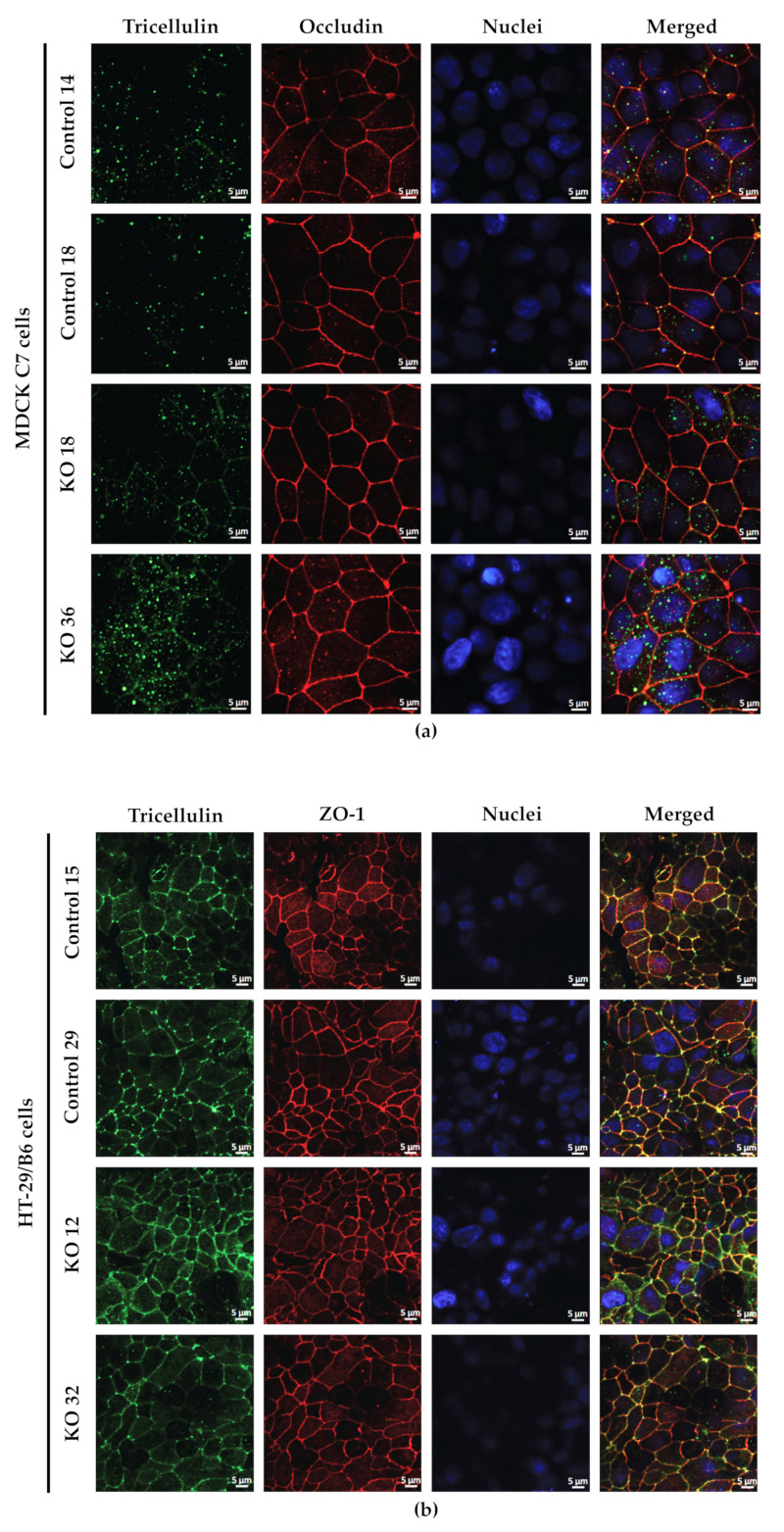
Localization of tricellulin in control and angulin-1 knockout cells. (**a**) Immunostaining of tricellulin in MDCK C7 angulin-1 KO cells. After angulin-1 knockout, tricellulin was still located in the tTJ, but additionally in the bTJ. (**b**) Immunostaining of tricellulin in HT-29/B6 angulin-1 KO cells. After angulin-1 knockout, tricellulin was found in both tTJ and bTJ, similar to control cells.

It is known that angulin-1 knockout could have different side effects on the localization of other tight junction proteins, specifically tricellulin. Immunofluorescence studies in combination with confocal microscopy revealed a slight delocalization of tricellulin from the tTJs to the bTJ after angulin-1 knockout in both cell lines, whereas occludin was concentrated at the corners of tTJs (Figure 3). These data suggest that angulin-1 has a role in the localization of tricellulin in both cell lines, nevertheless it does not play an essential role in the localization of other TJ proteins (data not shown).

### 2.4. Effect of Angulin-1 Knockout on the TJ Ultrastructural Level in MDCK C7 and HT-29/B6 Cells

**Figure 4:**
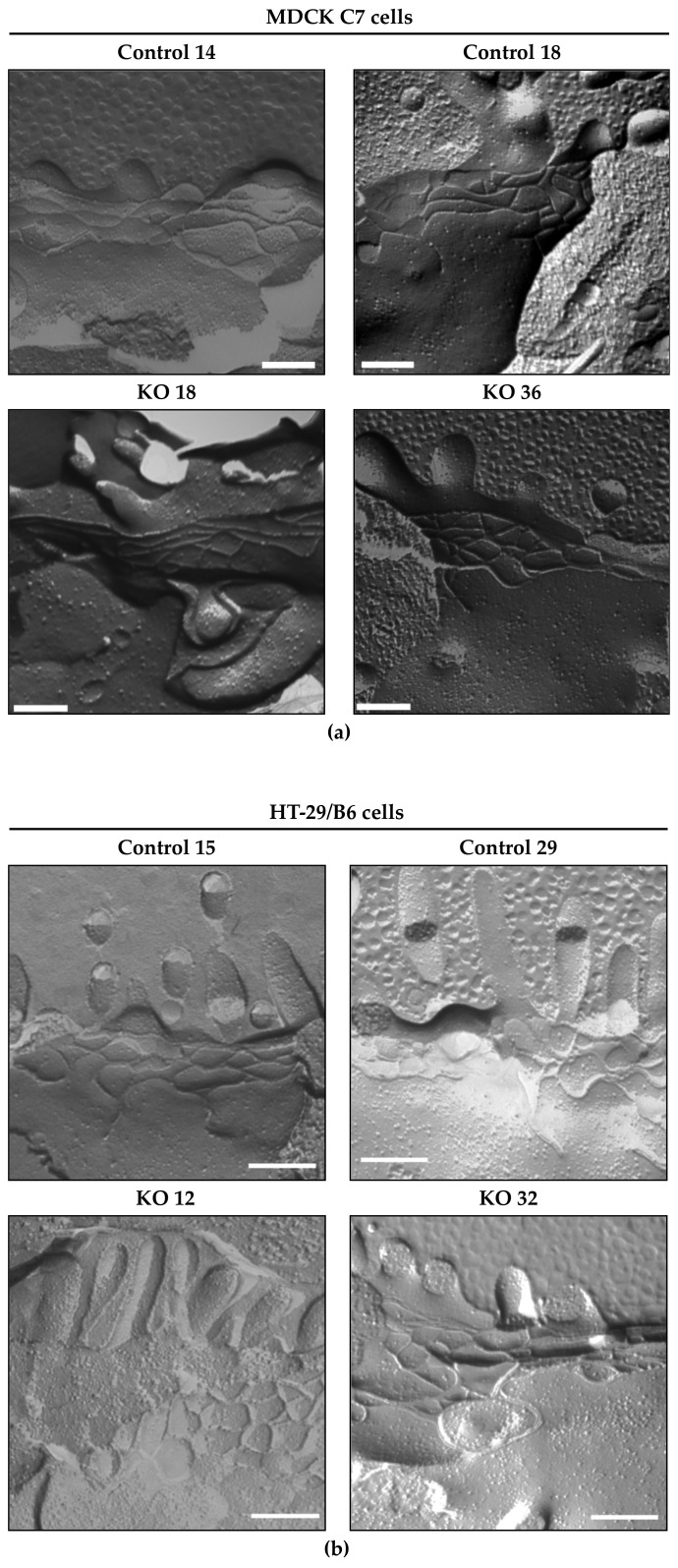
Freeze-fracture electron microscopy of angulin-1 knockout in (**a**) MDCK C7 and (**b**) HT-29/B6 cells. Photos were taken at ×51,000; Bars: 200 nm. The bTJ strands of the control cells and angulin-1 KO clones revealed a regular meshwork, characterized by continuous-type areas. bTJs of angulin-1 KO cells showed no ultrastructural difference compared with control clones in both cell lines.

To obtain insight of whether and to what extent angulin-1 influences the barrier properties of the TJ in MDCK C7 and HT-29/B6 cells, the ultrastructure of the TJs was analyzed by freeze-fracture electron microscopy (Figure 4). Although it would be interesting to investigate the tTJ structure in the angulin-1 KO cells, we could not find a sufficient number of tTJs in order to discuss the changes of the structure because the tTJs are quite rare and difficult to find in the freeze fracture replica samples.

Comparison of the bTJs (Figure 4a,b) of controls and angulin-1 KO clones showed no alteration in the ultrastructure and revealed a regular meshwork in both cell lines.

Regarding bTJs (Table 1), no alteration was found in the horizontally oriented filaments arranged perpendicular to the paracellular diffusion pathway between the controls and the angulin-1 knockouts in both cell lines. Neither the numbers of strands nor the meshwork depth was changed after angulin-1 KO (Table 1). The network density, which is calculated from the ratio of the number of strands to the network depth, did not differ between controls and angulin-1 knockout clones (Table 1). The number of breaks (>20 nm) per µm horizontal length of single-strands in bTJ was not significantly different between controls and the angulin-1 KO clones (Table 1). More importantly, analysis of strand appearance as being either of continuous- or particle- (pearl string) type revealed no changes that correlated with the observations reported above. Continuous strands appeared in all examined microscopy fields in control clones as well as in angulin-1 KO clones (Table 1). In addition, the TJs of the MDCK C7 controls and the angulin-1 KO clones were composed exclusively of linear strands; however, the HT-29/B6 control 29 and the angulin-1 KO 32 clones showed a slightly curved pattern (Table 1).

### 2.5. Effects of Angulin-1 Knockout on Ion Permeability in MDCK C7 and HT-29/B6 Cells

To examine whether the loss of angulin-1 affects the epithelial barrier development, the transepithelial resistance (TER), reflecting inverse overall ion permeability, was measured on controls and knockout clones.

**Figure 5:**
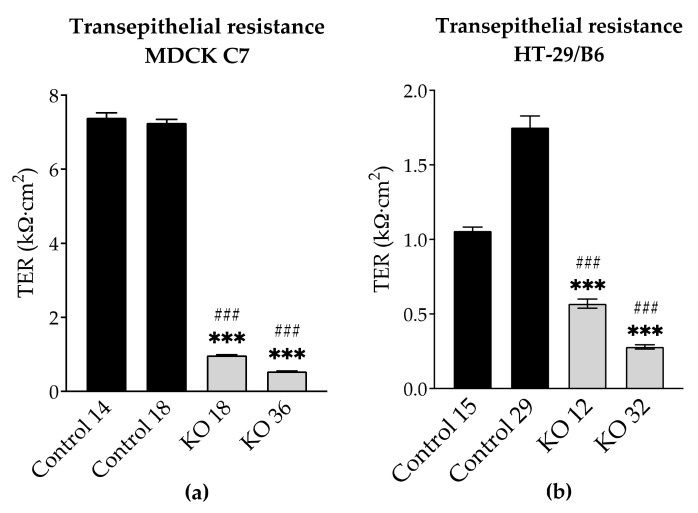
Functional analysis of angulin-1 knockout in MDCK C7 and HT-29/B6 cells. Effect of angulin-1 knockout on transepithelial resistance (TER) in (**a**) MDCK C7 (*n* = 50) and (**b**) HT-29/B6 cells (*n* = 43). Angulin-1 knockout strongly reduced TER in both cell lines. Statistical analysis was performed using one-way ANOVA test was used to compare between the four clones in both cell lines. (MDCK C7: *** *p* ≤ 0.001 with regard to control 14 and ^###^ *p* ≤ 0.001 with regard to control 18 and HT-29/B6: *** *p* ≤ 0.001 with regard to control 15 and ^###^ *p* ≤ 0.001 with regard to control 29).

TER was reduced in angulin-1 knockout clones in both cell lines (Figure 5a,b, Supplementary Tables S1 and S2). Angulin-1 KO caused a stronger decrease of TER in MDCK C7 cells than in HT-29/B6 cells (7 to 14 times in MDCK C7 cells and 2 to 7 times in HT-29/B6 cells).

### 2.6. Effects of Angulin-1 Knockout on Macromolecule Permeability in MDCK C7 and HT-29/B6 Cells

**Figure 6:**
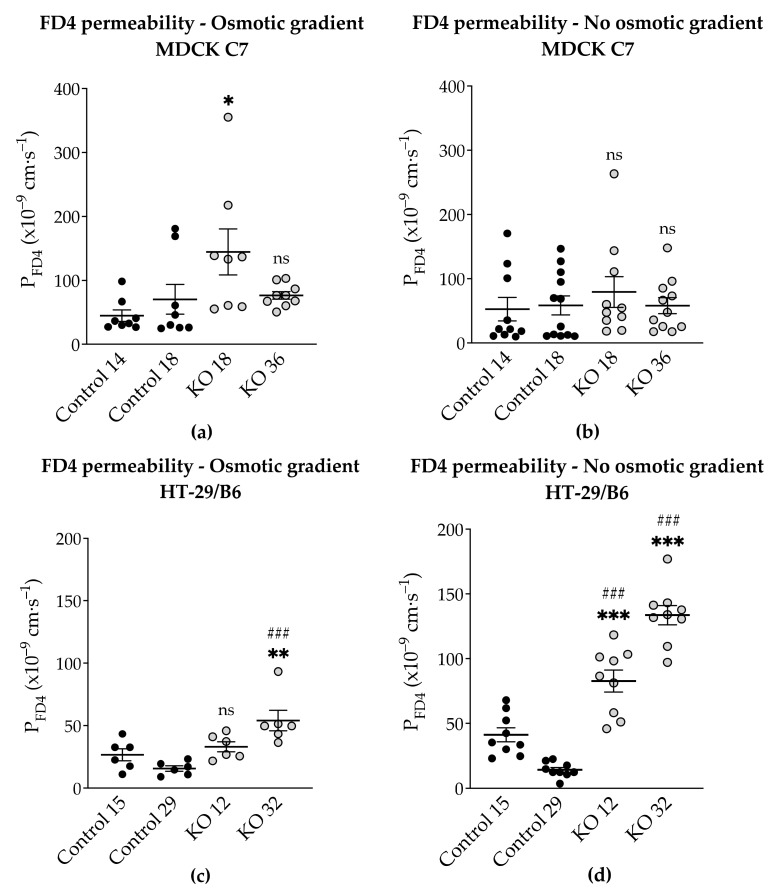
Functional analysis of angulin-1 knockout in MDCK C7 and HT-29/B6 cells. Effect of angulin-1 knockout on permeability for 4-kDa FITC-dextran (FD4) in MDCK C7 cells under an (**a**) osmotic gradient and (**b**) isosmotic condition. Permeability was increased only in the KO 18 clone under an osmotic gradient (*n* = 8–10). Effect of angulin-1 knockout on permeability for FD4 in HT-29/B6 cells under an (**c**) osmotic gradient and (**d**) isosmotic condition. Permeability was increased in both knockout clones under isosmotic conditions (*n* = 6–9), under osmotic gradient only the KO 32 clone showed an increase in permeability. Statistical analysis was performed using one-way ANOVA test was used to compare between the four clones in both cell lines (ns: not significant, MDCK C7: * *p* ≤ 0.05 with regard to control 14 and HT-29/B6: ** *p* ≤ 0.01, *** *p* ≤ 0.001 with regard to control 15 and ^###^ *p* ≤ 0.001 with regard to control 29).

The next question was whether angulin-1 knockout also affected the permeability to macromolecules. Therefore, we examined the permeability of the different clones to FITC-dextran 4 kDa (FD4) in two different conditions: (1) under an osmotic gradient or (2) under isosmotic conditions. As a result, FD4 permeability exhibited a strong increase in the angulin-1 knockout in HT-29/B6 cells specifically under isosmotic conditions (Figure 6c,d, Supplementary Table S2). In contrast, angulin-1 knockout did not increase the permeability to FD4 in MDCK C7 cells under isosmotic conditions (Figure 6b, Supplementary Table S1). Interestingly, under an osmotic gradient, only the KO 18 clone in MDCK C7 cells showed a slightly increase in FD4 permeability (Figure 6a, Supplementary Table S1). In MDCK C7 cells, there was no difference between FD4 permeability measured under osmotic and isosmotic conditions, whereas in HT-29/B6 cells, the FD4 permeability measured under isosmotic conditions was higher than measured under osmotic conditions.

### 2.7. Effect of Angulin-1 Knockout on Transepithelial Water Transport in MDCK C7 and HT-29/B6 Cells

**Figure 7:**
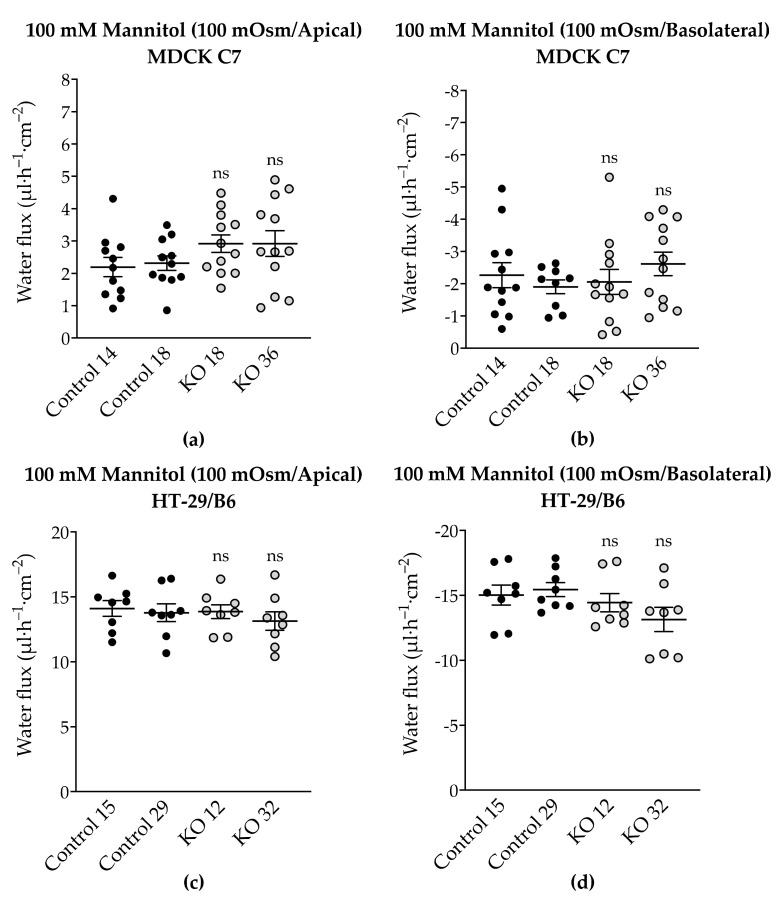
Water flux in control and angulin-1 knockout cells stimulated by an osmotic gradient on the apical or the basolateral side. Water flux induced by a gradient of 100 mM mannitol in (**a**,**b**) MDCK C7 and in (**c**,**d**) HT-29/B6 cells was unchanged. Statistical analysis was performed using one-way ANOVA test was used to compare between the four clones in both cell lines (ns: not significant).

To analyze the effect of angulin-1 knockout on water permeability of MDCK C7 and HT-29/B6 cells, water flux was measured after induction with an osmotic gradient produced by 100 mM mannitol either in the apical or the basolateral side of the cell layers (Figure 7, Supplementary Tables S1 and S2). This concentration produced measured osmolality gradients of 100 mOsm for 100 mM mannitol.

Surprisingly, and in contrast to what was previously found after tricellulin knockdown in MDCK C7 cells, angulin-1 knockout did not significantly change the water flux under the osmotic gradient induced by 100 mOsm mannitol (Figure 7a,b) compared with their respective controls. In the same way, in HT-29/B6 angulin-1 KO cells, water flux did not significantly change under the osmotic gradient induced by mannitol (Figure 7c,d) in comparison with their controls.

**Figure 8:**
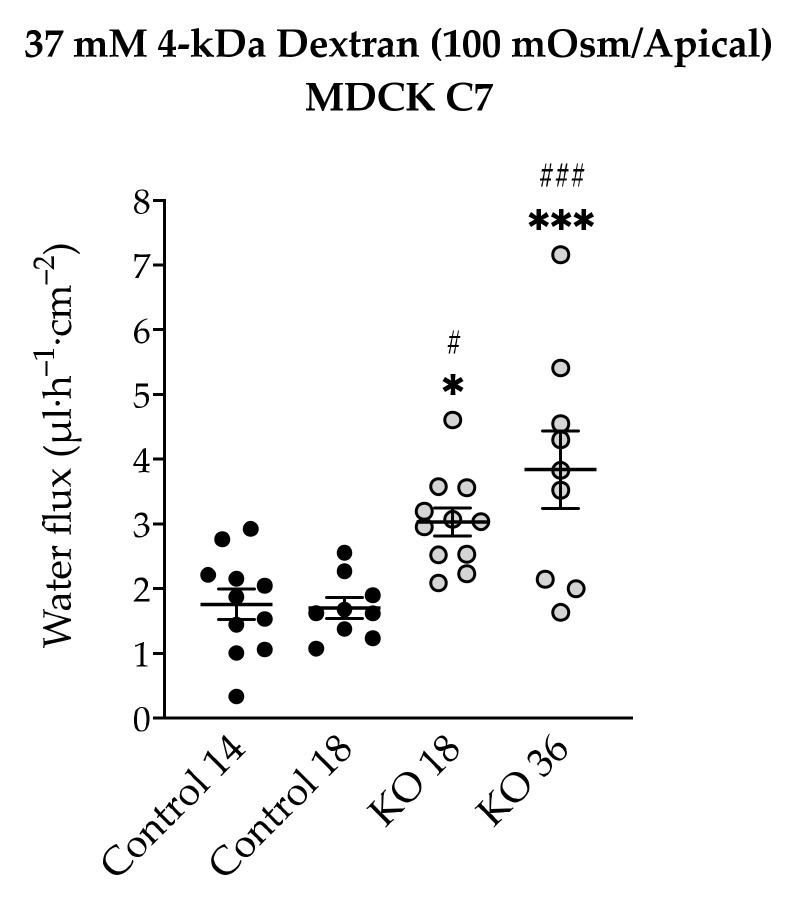
Water flux in angulin-1 knockout MDCK C7 cells induced by a gradient of 37 mM 4-kDa dextran on the apical side (*n* = 10–11). The transepithelial water transport increased in both angulin-1 knockout clones. (* *p* ≤ 0.05, *** *p* ≤ 0.001 with regard to control 14 and ^#^ *p* ≤ 0.05, ^###^ *p* ≤ 0.001 with regard to control 18).

In a parallel series of experiments, the water flux in the MDCK C7 angulin-1-depleted clones was measured after induction with an osmotic gradient produced by 37 mM 4-kDa dextran on the apical side because as we pointed out before, mannitol could go through the tTJ and interact with the pore or with the water molecules blocking their movement in the other direction. The water flux increased in both KO clones if 37 mM 4-kDa dextran (100 mOsm/Apical side) was used (Figure 8). This indicates that removal of angulin-1 from the tTJ altered the flux of water in a tight epithelium and that it is dependent on the chemical nature of the osmotic gradient.

**Figure 9:**
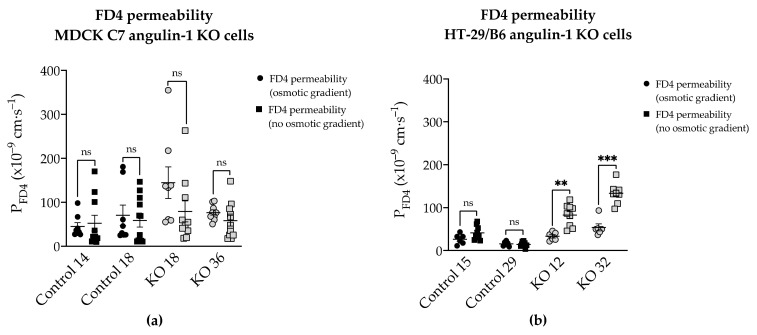
Comparison of angulin-1 knockout effect on permeability for 4-kDa FITC-dextran (FD4) under isosmotic and osmotic gradient conditions in (**a**) MDCK C7 and (**b**) HT-29/B6 cells. The FD4 permeability was higher under an isosmotic condition than under an osmotic gradient in angulin-1 KO HT-29/B6 cells, whereas no differences were observed in angulin-1 KO MDCK C7 cells. (ns: not significant, ** *p* ≤ 0.01, *** *p* ≤ 0.001).

As shown in Figure 9, there were differences in FD4 permeability under isosmotic and osmotic gradient conditions between both angulin-1 KO cell types, but there was no difference in FD4 permeability in MDCK C7 control and angulin-1 KO cells under both osmotic conditions. In contrast, in HT-29/B6 cells, no difference in control cells was found, except for an increase in FD4 permeability without any osmotic gradient (Figure 9). A possible explanation for this puzzling finding would be an interaction between the movement of water in the opposite direction to the movement of dextran under osmotic conditions in the tTJ of angulin-1 KO HT-29/B6 cells. This interaction appears not to exist in MDCK C7 angulin-1 KO cells.

### 2.8. Effect of Tricellulin KD on Transepithelial Water Transport in HT-29/B6 Cells

Previously, we described that in MDCK C7 cells tricellulin knockdown increased the transepithelial water flux compared to controls under different osmotic gradients [21]. In order to find out whether or not tricellulin has a similar effect in HT-29/B6 cells, tricellulin KD clones were generated and characterized in a similar way as we did for the MDCK C7 tricellulin KD clones (Table 2, Supplementary Figures S1–S4). Tricellulin KD in HT-29/B6 cells reduced the TER and increased the macromolecule permeability (Supplementary Figure S2). Interestingly, in tricellulin KD clones, the cation and water channel claudin-2 was upregulated (Supplementary Figure S3); however, it was not exclusively localized in the TJ, but also subjunctionally and intracellularly (Supplementary Figure S4a), and therefore this change did not affect ion and water permeability. In concordance with this, the ratio P^Na^/P^Cl^ did not change (Supplementary Figure S4b,c), which means that tricellulin increases the movement of ions without charge selectivity.

**Figure 10:**
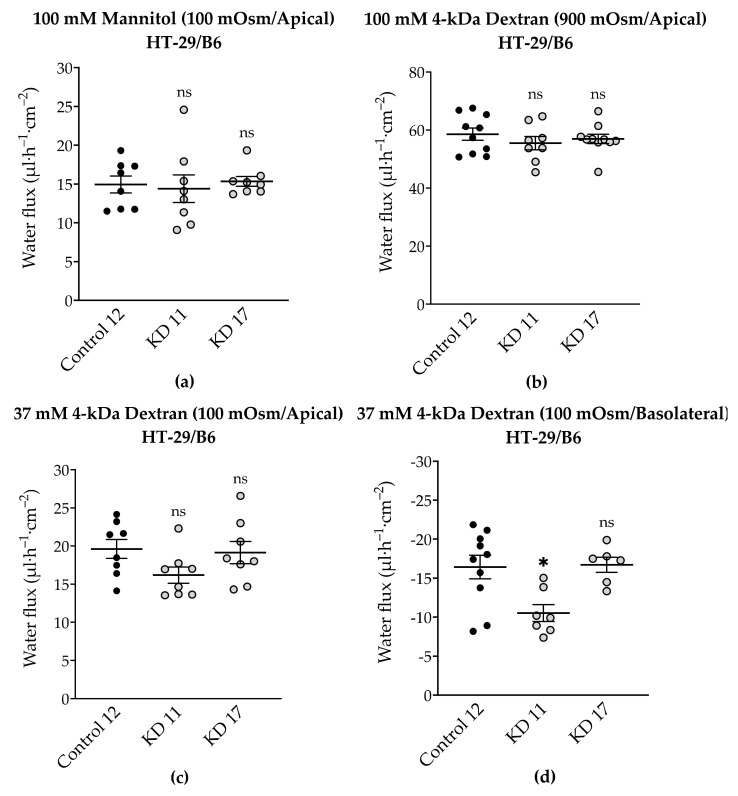
Water flux in control and tricellulin knockdown HT-29/B6 cells stimulated by an osmotic gradient. (**a**) Water flux induced by a gradient of 100 mM mannitol on the apical side. (**b**) Water flux induced by a gradient of 100 mM 4-kDa dextran on the apical side. (**c**) Water flux induced by a gradient of 37 mM 4-kDa dextran gradient on the apical side. (**d**) Water flux induced by a gradient of 37 mM 4-kDa dextran gradient on the basolateral side of the cell layer. (*n* = 8–10, ns: not significant, * *p* ≤ 0.05).

Regarding tricellulin, its knockdown did not regulate the transepithelial water transport through the tTJ in HT-29/B6 cells (Figure 10). In HT-29/B6 cells claudin-2 is genuinely expressed and a large component of water transport may have travelled through the bicellular TJ so that a possible contribution of the tTJ may not have reached significant levels.

## 3. Discussion

Tricellulin and angulin family proteins have been identified as molecular constituents of tTJs [5,22,25]. Tricellulin and angulins localize along the central elements of tTJs [5,25]. As angulins recruit tricellulin to TJs, the angulin–tricellulin axis is proposed to be of importance in tTJ formation [22,25,36]. Therefore, a dysregulation of angulins may critically affect the permeability for molecules up to 10 kDa in different epithelial organs.

In the present study, our focus was placed on elucidating the role of angulin-1 on paracellular water permeability. For this, the effect of angulin-1 knockout was analyzed in two different cell lines, MDCK C7, a tight epithelial cell line, and HT-29/B6, an intermediate-tight epithelial cell line. It turned out that angulin-1 is involved in water permeability only in the tight epithelial cell line (similar to what we previously reported on tricellulin [21]), while both angulin-1 and tricellulin have no effect on water permeability in an intermediate-tight epithelium. These results indicate that in tight epithelia, the tTJ is also essential for maintaining a barrier for water.

### 3.1. Angulin-1 Knockout Alters the Expression and Localization of Other Proteins in MDCK C7 and HT-29/B6 Cells

The expression of several tightening claudins in MDCK C7 cells was reduced in angulin-1 knockout cells, suggesting these claudins were partly removed from the TJ strands in the absence of angulin-1, in contrast, claudins and tricellulin expression were increased after angulin-1 knockout in HT-29/B6 cells, suggesting that these claudins were additionally incorporated into TJ strands in the absence of angulin-1. Most important, in both angulin-1 KO cell lines, tricellulin was partly removed from the tTJ and was also detectable in the bTJ [25].

On one hand, angulin-1 knockout in MDCK C7 cells induced a decrease in the expression level of occludin, claudin-1 and -4 (Figure 2c). This might affect the barrier function and formation as evidenced by Kirschner et al. through different knockdowns of claudin-1 and -4, occludin, and ZO-1, causing increased paracellular permeability for ions and larger molecules [37]. Furthermore, the absence of occludin and claudin-1 in both KO clones prevents the specific localization of tricellulin at tricellular contacts and promotes its localization at the bTJ [38,39]. These observations suggested that the proteins functionally influence each other.

On the other hand, angulin-1 knockout in HT-29/B6 cells increased the expression of barrier-forming claudins [39] and tricellulin (Figure 2d). Therefore, it can be concluded that the observed clonal variation of the claudins and tricellulin in sum is balanced and provides a constant barrier function of the bTJ and tTJ. These results suggested that the effects of angulin-1 and tricellulin relocalization from tTJs to bTJs on epithelial barrier function are controversial [39]. Interestingly, claudin-2 was downregulated in the KO 12 clone and upregulated in the KO 32 clone and appeared not to affect the water permeability. In relation to transcellular water transport, the major aquaporins of human colon cell line were found unchanged or downregulated, this suggests that changes in transepithelial water permeability could be attributed to the removal of angulin-1 and unlikely due to aquaporins. Lastly, the expression of LI-cadherin was increased in both KO clones, this could affect the water permeability under our experimental conditions. It was shown that water transport was impaired under hypertonic conditions when LI-cadherin is reduced [40,41]. Therefore, LI-cadherin might be important for water reabsorption of the gut at intraluminal hypertonic conditions [40,41].

Interestingly, the expression of angulin-2 and -3 did not change after the knockout of angulin-1 as a form of compensation in both cell lines. It was shown that compensatory functions exist between the angulin family members, e.g., in the organ of Corti [42], the large intestine and kidney [31], but this seems to be not relevant in the cell lines used for this study.

### 3.2. Angulin-1 Knockout Did Not Alter the Ultrastructure of the Bicellular Tight Junction in MDCK C7 and HT-29/B6 Cells

In previous RNAi experiments, the loss of tricellulin led to an unstable ultrastructure of tight junction, which ultimately caused the junctional complex to collapse completely [5]. In both cell lines, the loss of angulin-1 did not modify or alter the bTJ compared to the vector control clones, the TJ ultrastructure remains unchanged after angulin-1 knockout in MDCK C7 and HT-29/B6 cells (Figure 4).

It is established that, depending on the presence of claudin-2, strand discontinuities appear or disappear [43]. In our study, no discontinuities appear even if claudin-2 is increased in the HT-29/B6 angulin-1 KO 36 clone, possibly due to the upregulation of tricellulin.

Thus, an increase in ion permeability in both cell lines and an increase in macromolecule permeability in HT-29/B6 cells could possibly be caused by the opening of the tTJ because the only difference between the controls and the angulin-1 KO clones was the width of the central tube. However, the morphometric parameters could not be analyzed because of the low abundance of the same structures in both cell lines. After considering all possibilities, it is concluded that the knockout of angulin-1 did not induce resolvable changes in the ultrastructure of bTJs and tTJs that could affect water transport measurements.

### 3.3. Angulin-1 Knockout Increases the Ion Permeability in MDCK C7 and HT-29/B6 Cells

By the knockout of angulin-1, also the pore pathway was addressed (Figure 5). Similar to what was observed in tricellulin knockdown cells, the reduction in the expression of angulin-1 resulted in a lowered transepithelial resistance (TER). It is important to note that in MDCK C7 cells the removal of angulin-1 resulted in the reduction of the expression of most claudins while in HT-29/B6 the effect was the opposite. Therefore, it could be said that the reduction in TER on HT-29/B6 cells is due only to the reduction in angulin-1; however, in MDCK C7 cells the effect could be intensified due to the downregulation of other tightening TJ proteins. In conclusion, the decrease of angulin-1 within the tTJ also leads in both cell lines to a drop in paracellular resistance, which reciprocally reflects the permeability for ions.

### 3.4. Angulin-1 Knockout Increases the Macromolecule Permeability Only in HT-29/B6 Cells

As it is known, knockdown of angulin-1 in the mouse mammary gland Eph4 epithelial cells increases the permeability to macromolecules between 332 Da and 40 kDa [22]. Considering the above, the leak pathway was assessed (Figure 6). The present data showed that in HT-29/B6, a removal of angulin-1 strongly regulates the permeability for 4-kDa macromolecules. Nevertheless, it might be surprising that the same phenotype, in MDCK C7 cells, only slightly increased the permeability for macromolecules in one of the KO clones (KO 18), probably because in this KO clone, the tricellulin delocalization from the tTJ was larger than in the KO 36 clone.

Considering that HT-29/B6 is an intermediate-tight epithelial cell line and that its tTJ contributes at least 38% to the total paracellular conductance, it can be said that, not only did it affect the paracellular passage of the small solutes, including inorganic ions, but also of the macromolecules [20]. However, the contribution of tTJ in MDCK C7 cells is not yet known, but it would be expected to be higher than in HT-29/B6 cells because this cell line is highly tight [20], and therefore the permeability to FD4 would be higher after angulin-1 knockout, but this was not the case. This is perhaps due to tricellulin which has an affinity for claudin-based TJ strands within the plasma membrane if not directed to tricellular contacts by angulins [44] and also because occludin was concentrated in the corners of tTJ after angulin-1 KO (Figure 1c) probably stabilizing this structure. One could assume that due to redistribution of tricellulin into the bTJ a slight enhancement of bTJ barrier properties could occur [44].

Additionally, the permeability to FD4 was measured in two different conditions after angulin-1 KO and, as can be seen, in MDCK C7 cells there were no differences between isosmotic and osmotic conditions (Figure 9a). Conversely, in HT-29/B6 cells it was observed that the permeability to FD4 is higher under isosmotic conditions than under an osmotic gradient. A possible explanation for this might be that under osmotic conditions water moves in the opposite direction and this inhibits the movement of 4-kDa FITC-dextran, therefore a lower FD4 permeability was measured (Figure 9b). This water movement through the tTJ is too small to be detected in the overall transepithelial water flux measured in HT-29/B6 cells.

### 3.5. Angulin-1 Knockout Increases Transepithelial Water Transport Only in MDCK C7 Cells

To complete the tTJ permeability pattern, the transepithelial water transport was measured in the angulin-1 KO clones of MDCK C7 and HT-29/B6 cells. As described earlier, while the KO clones caused a decrease in TER in both cell lines, only in HT-29/B6 cells did angulin-1 KO increase the permeability to a 4-kDa macromolecule. Interestingly, the angulin-1 KO did not increase the water flux driven by 100 mM mannitol in both cell lines (Figure 7) and the total amount of water was independent of the direction of the osmotic gradient. The decision to use 4-kDa dextran was taken due to the dispersion of the values found in MDCK C7 cells when mannitol was used as an osmotic gradient; however, the values obtained with HT-29/B6 cells were more reproducible and therefore their study with 4-kDa dextran was obviated. As result, the water flux increased in the angulin-1 KO clones compared with their controls in MDCK C7 cells when 37 mM 4-kDa dextran was used as osmotic gradient (Figure 8).

Remarkably, in the case of MDCK C7 cells, an increase in water transport was expected like that found after reducing the expression of tricellulin in tTJ [21]; however, this was not the case when mannitol was used possibly due to the small diameter of this molecule with respect to the 4-kDa dextran which would facilitate its movement through tTJ thereby inhibiting the water flux in the converse direction. It is important to keep in mind that the reduction in the expression of most barrier-forming claudins could have an influence on the water transport after angulin-1 KO in MDCK C7 cells. As demonstrated by immunofluorescence confocal laser-scanning microscopy, tricellulin was redistributed from tTJ to bTJ, which could maintain and strengthen the epithelial barrier after the downregulation of several claudins. It is also important to note that the water fluxes measured in angulin-1 KO cells were lower than those measured in our previous work on tricellulin KD clones [21], demonstrating once again the importance of tricellulin in tTJ (for instance, angulin-1 KO 36 showed higher tricellulin displacement leading to higher water flux). In addition, it can be concluded that the reduction of tightening claudins did not increase the water flux in the KO clones with respect to their controls.

This phenomenon was also observed in HT-29/B6 cells; however, here the regulation of TJ expression was the opposite to what we observe in MDCK C7 cells, several claudins were upregulated, nevertheless, and no change in water transport could be measured. As shown in Figure 9, FD4 permeability in HT-29/B6 angulin-1 KO cells (not in control cells) measured under osmotic conditions is lower than under isosmotic conditions, potentially due to an opposite water flow across the tTJ. This means that the water flow across the tTJ, which was induced by a gradient using mannitol or dextran, could also be inhibited by the opposite passage of the osmotically active substances across the tTJ in these cells. This could also be the case in MDCK C7 angulin-1 KO cells when using mannitol for producing the osmotic gradient. In addition, the strong upregulation of tightening claudins in the bTJ in HT-29/B6 angulin-KO cells could diminish the water flux across the bTJ and thus mask a slight increase in water flux across the tTJ. Thus, we cannot completely exclude an increased water flow across the tTJ after downregulation of tricellulin or knockout of angulin-1, but this does not significantly contribute to the overall large water flow across an intermediate tight epithelial cell line like the HT-29/B6 cells.

In summary, the present study suggests a functional difference between angulin-2 and angulin-1 on the paracellular water transport via tTJs, which is an interesting issue to be pursued for the understanding of variations of tTJs. The effects of angulin-1 knockout on the epithelial barrier function vary by cell type and according to the interdependence between different TJ species affecting cell expression, localization and renewal. The reason could be the differences in angulin-1 interaction partners in bTJs and tTJ [22,39]. Therefore, combinations of proteins influenced by angulin-1 knockout in bTJs and tTJ vary by cell type and tissue, and the resulting epithelial barrier function of the mammalian epithelial cell sheet causes cell specific changes.

### 3.6. Tricellulin Knockdown Does Not Significantly Affect Paracellular Water Transport in the HT-29/B6 Cell Line

As was previously done for tricellulin knockdown clones in MDCK C7 cells [21], two tricellulin clones were selected in HT-29/B6 cells that differ in the grade of tricellulin reduction (Supplementary Figure S1). While both KD clones caused a decrease in TER and an increase in permeability to a 4-kDa macromolecule, none of them increased the water flux driven by different osmotic gradients. This may be due, as already described for the HT-29/B6 angulin knockout cells, to an inhibition of the water flux across the tTJ by a backflow of the osmotically active substances like mannitol or dextran. In addition, a large component of water transport may have traveled through the bTJ and a possible contribution from the tTJ may not have reached significant levels (Figure 10). On the other hand, there may be a threshold in the expression of tricellulin, that must be overcome to find significant differences in water transport, that were not reached with the knockdowns generated in this research. In addition, there is a possibility that in general the paracellular water flow is lower than the transcellular water flux or that there is a claudin-2-independent paracellular pathway for water.

In conclusion, in HT-29/B6 cells (intermediate-tight) the contribution of tricellulin to transepithelial water permeability is negligible, in contrast to MDCK C7 cells (tight) where tricellulin was able to regulate osmotically induced transepithelial water flux.

## 4. Materials and Methods

### 4.1. Cell Culture, Transfection and TER Measurement

The kidney cell line MDCK C7 (RRID: CVCL_0423) exhibits a high transepithelial resistance and other basic properties (no claudin-2 expression) making it an excellent model of a tight epithelium, and the intestinal cell line HT-29/B6 (RRID: CVCL_LJ30) exhibits a basic properties (moderate transepithelial resistance, low claudin-2 expression) making it an excellent model of an intermediate-tight epithelium. For stable angulin-1 knockout, MDCK C7 and HT-29/B6 cells were transfected with three sgRNA-CRISPR/ Cas9 vectors containing sequences targeting different exons of angulin-1 and corresponding HDR plasmids for direct homologous repair or sgRNA-CRISPR/Cas9 negative control (Santa Cruz, Heidelberg, Germany). For stable tricellulin knockdown, HT-29/B6 cells were transfected with pLKO.1-puro vector containing a sequence for shRNA targeting tricellulin (tricellulin shRNA; Sigma-Aldrich, Schnelldorf, Germany) or pLKO.1-puro empty vector as a negative control (Sigma-Aldrich, Schnelldorf, Germany).

The transfected cells were incubated at 37 °C and 5% CO_2_ in sterilization incubators held. For the cultivation of the cells sterile culture vessels made of plastic were used. For MDCK C7 knockout cells, a nutrient medium Earl’s salts MEM (minimal essential medium) supplemented with 10% FBS as well as 100 U/mL penicillin/100 μg/mL streptomycin and 1.5 µg/mL of puromycin was used. For HT-29/B6 knockdown and knockout cells, RPMI-1640 with stable L-glutamine medium supplemented with 10% FBS as well as 100 U/mL penicillin/100 μg/mL streptomycin and 1.5 µg/mL of puromycin was used. Every second to third day the medium was changed. Consumption of nutrients was also visible due to a color change of the medium contained a detecting pH indicator.

For protein quantification, water flux measurements and electrophysiological studies, cell monolayers were cultured on porous culture plate inserts (Millicell PCF filters, pore size 0.4 µm, effective area 0.6 cm^2^, Millipore GmbH, Schwalbach, Germany) for 7–10 days before they were used for experiments.

Transepithelial resistance (TER) was measured at 37 °C using chopstick electrodes (STX2, World Precision Instruments, Friedberg, Germany). Electrodes were reproducibly positioned by a semi-automatic motor-driven device and signals were processed by a low-frequency clamp (both own design). The resistances of the bathing solution and the blank filter support were subtracted from measured values, which were finally converted to Ω·cm^2^ [45].

### 4.2. Western Blot Analysis

Western Blot analysis was carried out as described before [21]. Cells grown on culture-plate inserts were scraped and homogenized in total lysis buffer containing 10 mM of Tris, 150 mM of NaCl, 0.5% of Triton X-100, and 0.1% of sodium dodecyl sulphate (SDS), and protease inhibitors (cOmplete^TM^ EDTA free; Roche, Basel, Switzerland). The samples were incubated for 2 h at 4 °C (vortexed every 20 min), and then centrifuged at 11,000× *g* during 20 min. The pellet was discarded and the protein concentration in the supernatant was determined by the BCA (bicinchoninic acid) method (reagents were purchased from Pierce (Perbio Science, Bonn, Germany)) and quantified with a plate reader (Tecan Deutschland, Crailsheim, Germany). After this, samples were prepared with a loading buffer containing 100 mM Tris-HCl (pH 6.8), 2% SDS, 10% glycerol, 100 mM DTT and 0.001% bromophenol blue. Each sample was then boiled at 95 °C for 10 min before loading onto the gel.

Aliquots between 10 and 15 µg protein samples were separated by 12% SDS-polyacrylamide gel electrophoresis and then transferred to a PVDF membrane (Perkin Elmer, Rodgau, Germany) for detection of angulins, TAMPs, claudins, AQPs, SGLT-1 and LI-cadherin. After blocking for 2 h in 1% PVP-40 and 0.05% Tween-20, membranes were incubated overnight with primary antibodies specific for angulin-1 (Sigma-Aldrich, Taufkirchen, Germany), angulin-2 (Aviva Systems Biology, San Diego, CA, USA), and angulin-3 (Sigma-Aldrich, Taufkirchen, Germany), tricellulin (Thermo Fisher Scientific, Invitrogen, Darmstadt, Germany), occludin (Thermo Fisher Scientific, Invitrogen, Darmstadt, Germany), claudin-1, -2, -3, -4, -5, -7, and -8 (Thermo Fisher Scientific, Invitrogen, Darmstadt, Germany), AQP-1 (OriGene Technologies, Herford, Germany), AQP-3, -4 and -7 (Santa Cruz, Heidelberg, Germany), SGLT1 (LSBio, Corston, UK) and LI-cadherin (Santa Cruz, Heidelberg, Germany).

After removing the first antibody and three washing steps, the membranes were incubated for 2 h with the second peroxidase-conjugated antibody (anti-mouse or anti-rabbit) in 1.5% of milk powder prepared in TBST 1X. The primary antibodies were used in dilutions of 1:1000 with the exception of AQP antibodies (1:1500), tricellulin (1:2000), and angulin-1 (1:3000). The secondary antibodies were used in dilutions of 1:1000. For detection of the chemiluminescence signal induced by addition of Lumi-LightPLUS Western blotting kit (Roche, Mannheim, Germany) a Fusion FX7 (Vilber Lourmat, Eberhardzell, Germany) was used. Densitometric analysis was performed with a quantification software (Image Studio™ Lite, LI-COR Biosciences, Lincoln, NE, USA). Equal protein loading in each lane was verified by comparison with signals for β-actin (Sigma-Aldrich, Taufkirchen, Germany). For Western blot analysis, lysates of at least three individual cell cultures were used and one representative experiment is shown.

### 4.3. Immunofluorescent Staining

Immunofluorescence analysis was carried out as described before [21]. Immunofluorescence studies were performed on culture-plate inserts. Confluent monolayers were rinsed with PBS, fixed with 4% paraformaldehyde for 20 min, washed three times with PBS, and permeabilized for 10 min with PBS containing 0.5% (*v*/*v*) Triton X-100. To block non-specific binding sites, cells were then incubated in PBS containing 1% (*w*/*v*) BSA and 5% (*v*/*v*) goat serum (blocking solution; Biochrom) for 60 min. All subsequent washing procedures were performed with this blocking solution.

After blocking, cells were incubated overnight at 4 °C with primary antibodies for angulin-1 (1:1000; Sigma-Aldrich), tricellulin (1:500; Thermo Fisher Scientific, Invitrogen), occludin (1:250; Thermo Fisher Scientific, Invitrogen) and ZO-1 (1:250; Thermo Fisher Scientific, Invitrogen), followed by washing steps and incubation during 60 min at room temperature with the respective secondary antibodies (Alexa Fluor 488 goat anti-rabbit and Alexa Fluor 594 goat anti-mouse, each 1:500; Thermo Fisher Scientific, Waltham MA, USA) and 4′,6-diamidino-2-phenylindole (DAPI, 1:1000; Roche, Mannheim, Germany). Cell culture inserts were mounted on microscope slides using ProTag MountFluor (Biocyc, Luckenwalde, Germany). Images were obtained with a confocal laser-scanning microscope (LSM 780, Zeiss, Jena, Germany) and processed using ZEN software (Carl Zeiss, Oberkochen, Germany, ZEN black edition 2012 SP1, ver. 8.1).

### 4.4. Freeze Fracture Electron Microscopy

At a confluency of 100%, cells were washed with PBS (containing Ca^2+^/Mg^2+^) and fixed with 2.5% glutaraldehyde at RT for 2 h. After washing twice with PBS (containing Ca^2+^/Mg^2+^), the cells were stored at 4 °C in 0.25% glutaraldehyde. Small rectangles of the bottom of the cell culture filters were cut out, the attached cells were cryoprotected in 30% glycerol for 30 min, placed between two gold specimen holders and shock frozen in R422D (TEGA GmbH, Würzburg, Germany) cooled by liquid nitrogen (−210 °C). The samples were fractured using the freeze-fracture device Denton DV-502 (Denton Vacuum, Moorestown, NJ, USA) at −100 °C and 2 × 10^−10^ Torr. The samples were vaporized at −150 °C (2 × 10^−10^ Torr) with a layer of platinum and then a layer of carbon. This results in a thin metal film on the broken sample. The replicas were cleaned with 12% sodium hypochlorite, washed several times in ddH_2_O and mounted on a copper mesh grid. The Zeiss 902A electron microscope was used to examine the replicas at 80 kV. Magnifications between 20,000 and 50,000 was used.

### 4.5. Measurement of 4-kDa FITC-Dextran Flux

Flux studies were performed in conventional Ussing chambers for cell-culture inserts [45] under voltage clamp conditions. Dextran flux was measured in 5 mL circulating 111-Ringer’s ((in mM) 119.7 NaCl, 21.4 NaHCO_3_, 2.5 Na_2_HPO_4_, 0.6 NaH_2_PO_4_, 5.7 KCl, 1.3 MgCl_2_, 1.2 CaCl_2_, and 10.0 D(+)-glucose) containing also 37 mM unlabeled 4-kDa dextran (SERVA, Heidelberg, Germany) on both sides of the cells for isosmotic conditions and only on the apical side for osmotic conditions. After addition of 0.2 mM 4-kDa FITC-labeled dialyzed dextran (Sigma-Aldrich) to the apical chamber (final concentration), basolateral samples (200 μL) were collected at 0-, 20-, 40-, 60-, 80-, 100-, 120- and 140-min. Tracer fluxes were determined from FITC-dextran samples, which were measured with a fluorometer at 520 nm (Spectramax Gemini, Molecular Devices, Ismaning, Germany). Dextran permeability was calculated from *p* = J/Δc with *p* = permeability (cm·s^−1^), J = flux (mol·h^−1^·cm^−2^) and c = concentration (mol/L).

### 4.6. Dilution Potential Measurements

Dilution potential measurements for the determination of ion permeabilities were performed in Ussing chambers for cell-culture inserts [45]. Water-jacketed gas lifts kept at 37 °C were filled with 10 mL circulating fluid on each side. The bathing solution contained (in mM) 119.7 NaCl, 21.4 NaHCO_3_, 5.7 KCl, 1.3 MgCl_2_, 1.2 CaCl_2_, 3.0 HEPES, and 10.0 D(+)-glucose, and was gassed with 95% O_2_ and 5% CO_2_ to ensure a pH value of 7.4. All experimental data were corrected for the resistance of the empty filter and the bathing solution. Dilution potentials were measured with modified bathing solution on the apical or basolateral side of the epithelial monolayer. In the modified bathing solution, NaCl was iso-osmotically replaced by mannitol. The ratio of P^Na^ and P^Cl^ and the absolute permeabilities for Na^+^ and Cl^−^ were calculated as described before [46].

### 4.7. Measurement of Transepithelial Water Transport

Water flux measurements were performed using a modified Ussing chamber developed by our group [12,13,14,21]. The gas lifts of the common Ussing chamber were replaced by two glass tubules with a small diameter, where the menisci of the perfusion solution are clearly visible. From changes in the menisci, the water flux is calculated. Throughout these experiments, transepithelial voltage (mV) was clamped to 0 mV and transepithelial resistance (TER, Ω·cm^2^) and short-circuit current (I_SC_, μA·cm^−2^) were recorded. Resistances of bathing solution and blank filter support were measured prior to each experiment and subtracted. The stability of TER served as an indicator of cell viability.

Cell filters were mounted in Ussing chambers and perfused with HEPES-buffered solution with the following composition (in mM): 144.8 NaCl, 2.4 Na_2_HPO_4_, 0.6 NaH_2_PO_4_, 5.4 KCl, 1.2 MgCl_2_, 1.2 CaCl_2_, 10.6 HEPES, and 10.0 D(+)-glucose. The pH value of the perfusion solution was pH 7.4. A rotary pump ensured constant circulation of the perfusion solution (4.0 mL·min^−1^) and thus a fast fluid exchange in both hemichambers (volume 500 μL) to avoid effects of unstirred layers on water permeability. Water flux was induced by a transepithelial osmotic gradient: (i) 100 mM mannitol, (ii), 37 mM 4 kDa-dextran or (iii) 100 mM 4 kDa-dextran. The solution was added in the apical or basolateral compartment of the Ussing chamber. The osmolality of the perfusion solutions (mosmol/kg, abbreviated mOsm) was determined using a freezing point depression osmometer (Osmomat 3000, Gonotec, Berlin, Germany). The osmolality of the HEPES-buffered solutions is annotated in Table 3.

The fluid level in both glass tubes was monitored by a visual system ColorView XS (Olympus Soft Imaging Solutions GmbH, Munster, Germany) at time 0 min and with intervals of 10 min over a period of 120 min. Transepithelial water flux, given as flux per square centimeter and hour, was calculated after special calibration from the difference between the menisci at the registration times. Fluxes directed from the basolateral to the apical compartment were defined as positive flux.

### 4.8. Statistical Analysis

Data are expressed as mean values ± SEM, indicating *n* as the number of single measurements, and N is the number of independent experiments, which means independent seeding of cells. Statistical analysis was performed using Student’s *t*-test between the tricellulin KD clones and their corresponding control (KD 11 and KD 17 versus control 12). In case of angulin-1 KO clones, statistical significance of the difference between means was evaluated using one-way ANOVA and the Tukey and Dunnett adjustment for multiple testing.

MDCK C7 cells: * *p* ≤ 0.05, ** *p* ≤ 0.01, *** *p* ≤ 0.001 with regard to control 14 and ^#^ *p* ≤ 0.05, ^##^ *p* ≤ 0.01, ^###^ *p* ≤ 0.001 with regard to control 18. HT-29/B6 cells: * *p* ≤ 0.05, ** *p* ≤ 0.01, *** *p* ≤ 0.001 with regard to control 15 and ^#^ *p* ≤ 0.05, ^##^ *p* ≤ 0.01, ^###^ *p* ≤ 0.001 with regard to control 29. Statistical analysis was performed using GraphPad Prism 8 software (GraphPad; San Diego, CA, USA). A probability value of *p* ≤ 0.05 was considered statistically significant.

## 5. Conclusions

The major goal of this research was to clarify whether or not the expression of the tricellular tight junction (tTJ) protein, angulin-1, is involved in controlling paracellular water transport in cell lines with different levels of tightness (HT-29/B6 and MDCK C7 cells). This work supports the previous hypothesis that tTJs function differentially in distinct epithelial cell lines.

In brief, we provide insights into tTJ permeability by showing that angulin-1 can function as regulator of the macromolecular barrier in the tricellular tight junction regardless of the tightness of the epithelium (Supplementary Table S3). Manipulating the tTJ permeability (via tricellulin or via angulin-1) represents a mechanism to regulate epithelial water homeostasis only in tight epithelial cell lines (MDCK C7 cells); nevertheless, in intermediate-tight epithelial cell lines (HT-29/B6 cells) the contribution of tTJ seems to be negligible. Thus, the contribution of tTJ to transepithelial water transport depends on the tightness of the epithelium. Probably, then, the regulation of water paracellular transport by osmolytes in each type of epithelial cell has physiological significance depending on the organ to which it belongs.

## Figures and Tables

**Table 1:** Morphometric analysis of TJ ultrastructure of MDCK C7 and HT-29/B6 angulin-1 KO cells. No significant differences in TJ ultrastructure of control cells and angulin-1 KO cells could be detected using freeze-fracture electron microscopic analysis in MDCK C7 and HT-29/B6 cells.

		MDCK C7 Cells				HT-29/B6 Cells			
		Control 14 (*n* = 29)	Control 18 (*n* = 26)	KO 18 (*n* = 18)	KO 36 (*n* = 28)	Control 15 (*n* = 17)	Control 29 (*n* = 20)	KO 12 (*n* = 11)	KO 32 (*n* = 27)
Number of strands		3.62 ± 0.23	3.42 ± 0.20	4.17 ± 0.35	3.46 ± 0.23	4.53 ± 0.39	3.61 ± 0.34	3.91 ± 0.31	4.59 ± 0.41
Meshwork depth ^a^ (nm)		151.03 ± 11.00	165.00 ± 22.42	171.11 ± 24.78	157.50 ± 16.22	276.47 ± 37.42	228.89 ± 36.71	219.09 ± 23.06	310.93 ± 39.08
Strand density ^b^ (1/pm)		23.97 ± 2.32	20.75 ± 3.07	24.35 ± 4.09	22.00 ± 2.70	16.38 ± 2.64	15.78 ± 2.94	17.84 ± 2.36	14.77 ± 2.27
Number of breaks ^c^ per µm		0.0 ± 0.0	0.01 ± 0.009	0.0 ± 0.0	0.0 ± 0.0	0.02 ± 0.015	0.0 ± 0.0	0.03 ± 0.029	0.0 ± 0.0
Strand appearance	Continuous (%)	100	92	100	100	94	100	100	93
	Particle (%)	0	8	0	0	6	0	0	7
Strand pattern	Straight (%)	100	100	100	100	100	89	100	85
	Curved (%)	0	0	0	0	0	11	0	15

The meshwork depth ^a^ is defined as the distance between the apical and the contra-apical strand. Breaks ^c^ are defined as strand discontinuities >20 nm within the compact TJ meshwork; their number is given per μm length of horizontally oriented strands. The density ^b^ of bTJ (bicellular tight junction) strands is the ratio of strand number and meshwork depth and given in number per pm meshwork depth.

**Table 2:** Characteristics of HT-29/B6 tricellulin knockdown clones and the corresponding control. Two tricellulin knockdown clones and its corresponding control were analyzed in this study (Control 12, KD 11 and KD 17). Data of tricellulin expression have been obtained by densitometric analysis of Western blots using β-actin for normalization. Paracellular permeability measurements for FD4 were carried out in the Ussing chamber. Data of P^Na^/P^Cl^ permeability and absolute permeabilities for Na^+^ and Cl^−^ (P^Na^, P^Cl^) were obtained from dilution potential measurements in the Ussing chamber. Water flux measurements were performed in a modified Ussing chamber with water flux induced by different osmotic gradients.

		Control 12	KD 11	KD 17
Tricellulin expression (%)		100.0 ± 8.9 (*n* = 11)	64.6 ± 4.7 *** (*n* = 10)	55.7 ± 6.3 *** (*n* = 10)
TER (kΩ·cm^2^)		1.44 ± 0.07 (*n* = 12)	0.51 ± 0.03 *** (*n* = 12)	0.54 ± 0.02 *** (*n* = 12)
P_FD4_ (×10^−9^ cm·s^−1^) Osmotic gradient		21.34 ± 3.80 (*n* = 7)	42.96 ± 2.56 *** (*n* = 7)	67.55 ± 6.54 *** (*n* = 7)
P^Na^ (×10^−6^ cm·s^−1^)		0.61 ± 0.10 (*n* = 10)	1.50 ± 0.11 *** (*n* = 10)	2.46 ± 0.30 *** (*n* = 11)
P^Cl^ (×10^−6^ cm·s^−1^)		0.80 ± 0.18 (*n* = 10)	1.84 ± 0.17 ** (*n* = 10)	2.98 ± 0.33 ** (*n* = 11)
P^Na^/P^Cl^		0.91 ± 0.08 (*n* = 10)	0.92 ± 0.06 (*n* = 10)	0.90 ± 0.08 (*n* = 11)
Water flux—apical side (µL·h^−1^·cm^−2^)	100 mM mannitol (100 mOsm)	14.9 ± 1.0 (*n* = 8)	14.4 ± 1.7 (*n* = 8)	15.3 ± 0.6 (*n* = 8)
	37 mM 4-kDa dextran (100 mOsm)	19.6 ± 1.2 (*n* = 8)	16.2 ± 1.0 (*n* = 8)	19.1 ± 1.4 (*n* = 8)
	100 mM 4-kDa dextran (900 mOsm)	58.6 ± 2.0 (*n* = 10)	54.0 ± 2.4 (*n* = 9)	56.9 ± 1.6 (*n* = 10)
Water flux—basolateral side (µL·h^−1^·cm^−2^)	37 mM 4-kDa dextran (100 mOsm)	−16.4 ± 1.4 (*n* = 10)	−10.5 ± 1.0 * (*n* = 7)	−16.7 ± 0.9 (*n* = 6)

Significances refer to the control. *n* number of experiments, * *p* ≤ 0.05, ** *p* ≤ 0.01, *** *p* ≤ 0.001.

**Table 3:** Osmolality of the HEPES-buffered solutions using a freezing point depression osmometer.

		*n*	Concentration (mM)	Osmolality (mOsm/kg of Water) Mean ± SEM
Angulin-1 KO	HEPES only	17	-	289.0 ± 13.7
	+Mannitol	17	100 mM	391.4 ± 14.6
	+4-kDa dextran	5	37 mM	396.0 ± 4.5
Tricellulin KD	HEPES only	10	-	308.0 ± 4.4
	+Mannitol	10	100 mM	410.3 ± 1.9
	+4-kDa dextran	10	37 mM	396.2 ± 6.8
	+4-kDa dextran	10	100 mM	877.8 ± 13.7

## Data Availability

Data is contained within the article or Supplementary Materials.

## Supplementary Materials

Supplementary Materials can be found at https://www.mdpi.com/article/10.3390/ijms22157827/s1.

## Abbreviations

angulin-1	lipolysis-stimulated lipoprotein receptor (LSR)
angulin-2	immunoglobulin-like domain containing receptor-1 (ILDR1)
angulin-3	immunoglobulin-like domain containing receptor-2 (ILDR2)
bTJ	bicellular tight junction, located between two cells
CRISPR/Cas9	Clustered Regularly Interspaced Short Palindromic Repeats/Cas9
d(Å)	diameter (Å)
FD4	4-kDa FITC-dextran
HDR	homology-directed repair
KD	knockdown
KO	knockout
SEM	standard error of the mean
TJ	tight junction
TER	transepithelial resistance (Ω·cm^2^)
tTJ	tricellular tight junction, located at corners of three cells
P	Apparent permeability (cm·s^−1^)
