# Supplementary material for: Angulin-1 (LSR) Affects Paracellular Water Transport, However Only in Tight Epithelial Cells
Source: Int J Mol Sci. 2021 Jul 22;22(15):7827. doi: 10.3390/ijms22157827 (PMC8346120; doi:10.3390/ijms22157827)
Supplement: Supplementary file 1 [file ijms-22-07827-s001.zip › ijms-1262014-supplementary.pdf]

International Journal of *Molecular Sciences*

MDPI

Supplementary Materials

Part 1. Numerical data from angulin-1 knockout clones in MDCK C7 and HT-29/B6 cells.

Table S1. Characteristics of MDCK C7 angulin-1 knockout clones and the corresponding controls. Two angulin-1 knockout clones and their corresponding controls were analyzed in this study (Control 14, Control 18, KO 18 and KO 36). Data of angulin-1 expression have been obtained by densitometric analysis of Western blots using β-actin for normalization. Paracellular permeability measurements for FD4 were carried out in the Ussing chamber. Water flux measurements were performed in a modified Ussing chamber with water flux induced by different osmotic gradients.

		Control 14	Control 18	KO 18	KO 36
**Angulin-1 expression (%)**		99.2 ± 4.8 (*n*=9)	100.8 ± 5.1 (*n*=9)	0.10 ± 0.02 ***,### (*n*=9)	0.05 ± 0.01 ***,### (*n*=9)
**TER (kΩ·cm²)**		7.3 ± 0.2 (*n*=60)	7.4 ± 0.1 (*n*=60)	0.96 ± 0.02 ***,### (*n*=60)	0.53 ± 0.01 ***,### (*n*=60)
**P_{FD4} (×10⁻⁹ cm·s⁻¹) Osmotic gradient**		44.8 ± 8.3 (*n*=8)	70.4 ± 21.7 (*n*=8)	144.4 ± 33.6 * (*n*=8)	76.6 ± 5.5 (*n*=9)
**P_{FD4} (×10⁻⁹ cm·s⁻¹) Isosmotic**		52.4 ± 17.2 (*n*=10)	58.4 ± 14.1 (*n*=12)	79.3 ± 22.7 (*n*=10)	56.5 ± 12.8 (*n*=10)
**P_{D4} (×10⁻¹⁰ cm·s⁻¹) Osmotic gradient**		2.41 ± 0.45 (*n*=8)	3.79 ± 1.17 (*n*=8)	7.76 ± 1.81 * (*n*=8)	4.12 ± 0.30 (*n*=9)
**P_{D4} (×10⁻¹⁰ cm·s⁻¹) Isosmotic**		2.82 ± 0.93 (*n*=10)	3.14 ± 0.76 (*n*=12)	4.26 ± 1.22 (*n*=10)	3.12 ± 0.63 (*n*=11)
**Water flux – Apical side (µl·h⁻¹·cm⁻²)**	**100 mM mannitol (100 mOsm)**	2.20 ± 0.40 (*n*=7)	2.32 ± 0.30 (*n*=7)	2.92 ± 0.34 (*n*=8)	2.92 ± 0.56 (*n*=8)
**Water flux – Basolateral side (µl·h⁻¹·cm⁻²)**	**100 mM mannitol (100 mOsm)**	–2.27 ± 0.54 (*n*=8)	–1.90 ± 0.28 (*n*=6)	–2.07 ± 0.55 (*n*=8)	–2.62 ± 0.44 (*n*=8)

Significances refer to respective controls. *n* number of experiments, * $p \le 0.05$, ** $p \le 0.01$, *** $p \le 0.001$ with regard to control 14 and # $p \le 0.05$, ## $p \le 0.01$, ### $p \le 0.001$ with regard to control 18.

Table S2. Characteristics of HT-29/B6 angulin-1 knockout clones and the corresponding controls. Two angulin-1 knockout clones (KO 12 and KO 32) and their corresponding controls (Control 15, Control 29) were analyzed in this study. Data of angulin-1 expression have been obtained by densitometric analysis of Western blots using β-actin for normalization. Paracellular permeability measurements for FD4 were carried out in the Ussing chamber. Water flux measurements were performed in a modified Ussing chamber with water flux induced by different osmotic gradients.

		Control 15	Control 29	KO 12	KO 32
**Angulin-1 expression (%)**		94.3 ± 3.8 (*n*=9)	105.7 ± 5.4 (*n*=9)	3.2 ± 0.6 ***, ### (*n*=9)	0.2 ± 0.1 ***, ### (*n*=9)
**TER (kΩ·cm²)**		1.06 ± 0.02 (*n*=43)	1.75 ± 0.08 (*n*=43)	0.57 ± 0.03 ***, ### (*n*=43)	0.28 ± 0.01 ***, ### (*n*=43)
**P_{FD4} (×10⁻⁹ cm·s⁻¹) Osmotic gradient**		26.7± 4.4 (*n*=6)	15.7 ± 2.0 (*n*=6)	33.1 ± 3.6 (*n*=6)	54.0 ± 7.5 **, ### (*n*=6)
**P_{FD4} (×10⁻⁹ cm·s⁻¹) Isosmotic**		41.2 ± 5.1 (*n*=9)	14.1 ± 1.8 (*n*=9)	82.7 ± 8.0 ***, ### (*n*=9)	133.6 ± 7.0 ***, ### (*n*=9)
**P_{D4} (×10⁻¹⁰ cm·s⁻¹) Osmotic gradient**		4.28 ± 1.12 (*n*=6)	2.46 ± 0.57 (*n*=6)	4.85 ± 0.95 (*n*=6)	8.25 ± 2.00 # (*n*=6)
**P_{D4} (×10⁻¹⁰ cm·s⁻¹) Isosmotic**		4.08 ± 0.50 (*n*=9)	1.40 ± 0.18 (*n*=9)	8.19 ± 0.80 ***, ### (*n*=9)	13.23 ± 0.69 ***, ### (*n*=9)
**Water flux Apical side (µl·h⁻¹·cm⁻²)**	**100 mM mannitol (100 mOsm)**	14.1 ± 0.6 (*n*=8)	13.8 ± 0.6 (*n*=8)	13.9 ± 0.5 (*n*=8)	13.1 ± 0.7 (*n*=8)
**Water flux Basolateral side (µl·h⁻¹·cm⁻²)**	**100 mM mannitol (100 mOsm)**	–15.0 ± 0.7 (*n*=8)	–15.4 ± 0.5 (*n*=8)	–14.4 ± 0.7 (*n*=8)	–13.2 ± 0.9 (*n*=8)

Significances refer to respective controls. *n* number of experiments, * $p \le 0.05$, ** $p \le 0.01$, *** $p \le 0.001$ with regard to control 15 and # $p \le 0.05$, ## $p \le 0.01$, ### $p \le 0.001$ with regard to control 29.

Table S3. Barrier function and water permeability in the experimental cell models used in this work modulating tricellular tight junction proteins.

TJ protein	Method	Cell type	Observed changes in barrier function and water permeability
Tricellulin	Knockdown – shRNA	MDCK C7 [21]	TER:<br>KD 23 and KD 24 → Reduced
			Charge selectivity:<br>KD 23 and KD 24 → Unchanged
			4-kDa FITC-dextran flux:<br>KD 23 → Unchanged<br>KD 24 → Increased
			Upregulated proteins:<br>KD 23 → Claudin-1 and AQP-7
			Downregulated proteins:<br>KD 24 → Occludin, claudin-4 and -8
			**Transepithelial water permeability:<br>KD 23 and KD 24 → Increased**
		HT-29/B6	TER:<br>KD 11 and KD 17 → Reduced
			Charge selectivity:<br>KD 11 and KD 17 → Unchanged
			4-kDa FITC-dextran flux:<br>KD 11 → Increased<br>KD 17 → Increased
			Upregulated proteins:<br>KD 11 → Angulin-1, claudin-1, -2 and -8<br>KD 17 → Angulin-1, claudin-2, -3 and -8, AQP-4
			Downregulated proteins:<br>KD 17 → AQP-3
			**Transepithelial water permeability:<br>KD 11 and KD 17 → Unchanged**
Angulin-1	Knockout – CRISPR/Cas9/HDR	MDCK C7	TER:<br>KO 18 and KO 36 → Reduced
			4-kDa FITC-dextran flux:<br>KO 18 → Increased<br>KO 36 → Unchanged
			bTJ ultrastructure:<br>KO 18 and KO 36 → Unchanged
			Upregulated proteins:<br>KO 18 → AQP-1
			Downregulated proteins:<br>KO 18 → Occludin, claudin-1, -5, -7<br>KO 36 → Occludin, claudin-1, -3, -4, -5, -7, -8, AQP-7
			**Transepithelial water permeability:<br>KO 18 and KO 36 → Increased**
		HT-29/B6	TER:<br>KO 12 and KO 32 → Reduced
			4-kDa FITC-dextran flux:<br>KO 12 → Increased<br>KO 32 → Increased
			bTJ ultrastructure:<br>KO 12 and KO 32 → Unchanged
			Upregulated proteins:<br>KO 12 → Tricellulin, claudin-1, -5, -7 and -8, and LI-cadherin<br>KO 32 → Tricellulin, claudin-1, -3, -5, -7 and -8, and LI-cadherin
			Downregulated proteins:<br>KO 12 → Claudin-2, AQP-4 and SGLT1
			**Transepithelial water permeability:<br>KO 12 and KO 32 → Unchanged**

Part 2. Characterization of tricellulin KD clones in the HT-29/B6 cell line.

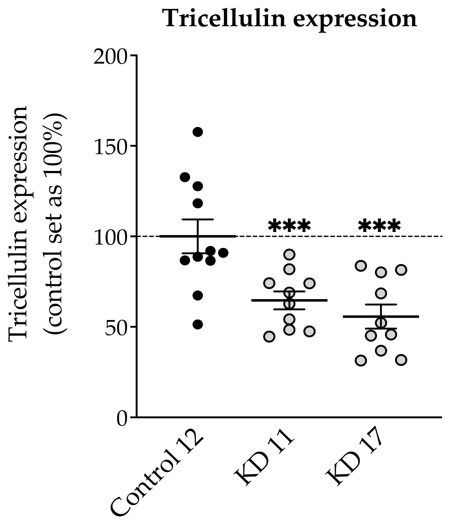

(a)

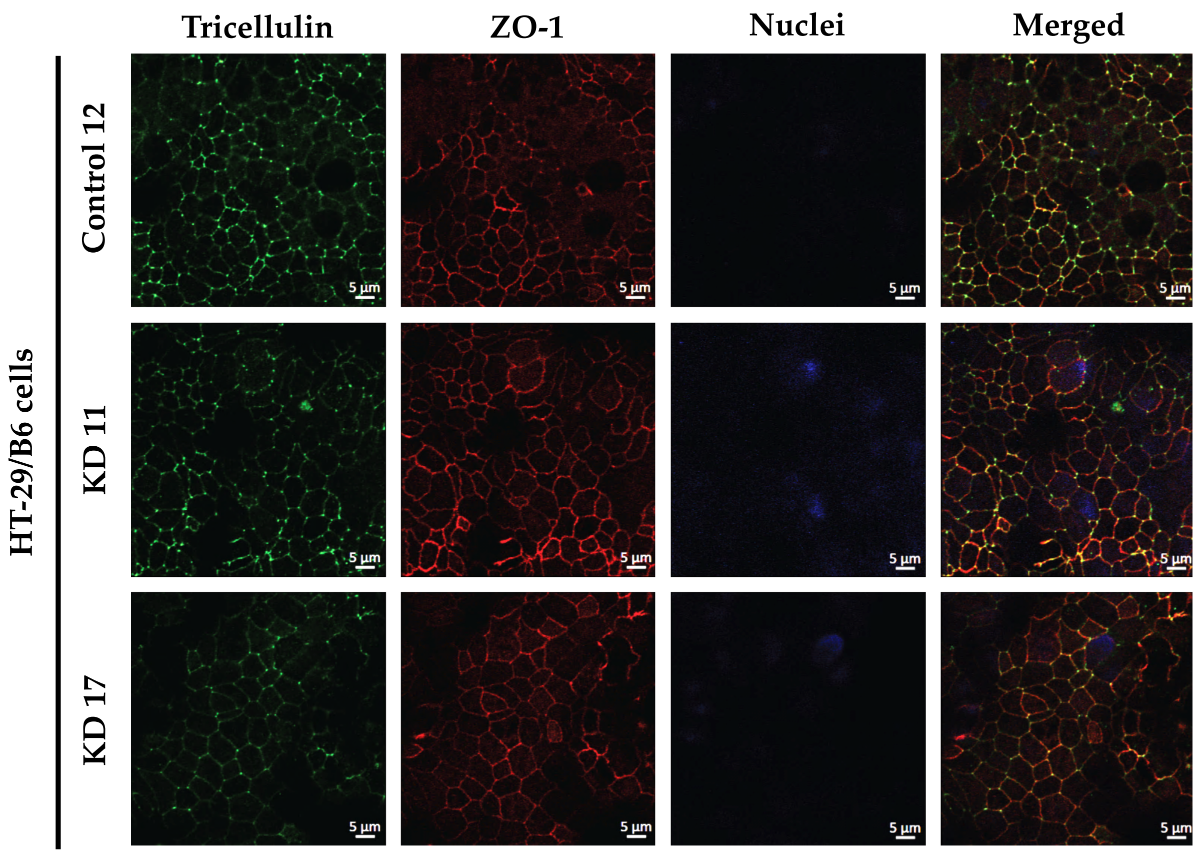

(b)

Figure S1. Expression and localization of tricellulin in HT-29/B6 cells. **(a)** Densitometric analysis of tricellulin protein expression levels in stable shTRIC transfectants in comparison to vector-transfected controls. shTRIC leads to decreased tricellulin expression (*** $p \leq 0.001$) and **(b)** Immunofluorescent staining of HT-29/B6 shRNA targeting tricellulin. Knockdown in HT-29/B6 cells had no effect on localization of tricellulin, which remained within the tTJ. Tricellulin: green, ZO-1: red, DAPI (nucleus): blue.

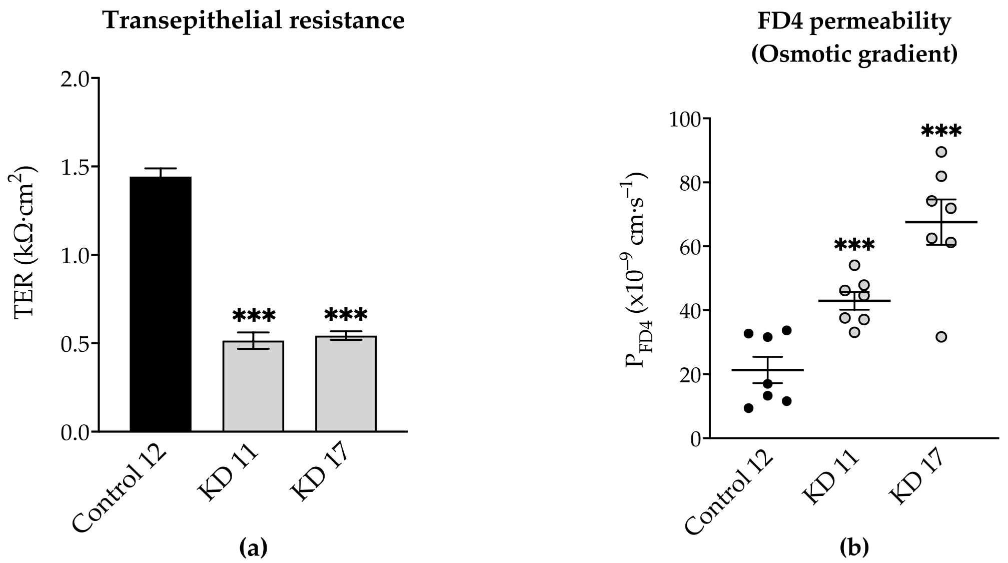

Figure S2. Functional analysis of tricellulin knockdown in HT-29/B6 cells. **(a)** Effect of tricellulin knockdown on transepithelial resistance. Tricellulin KD decreased TER in HT-29/B6 cells (n=24; *** $p \leq 0.001$). **(b)** Permeability to 4-kDa FITC-dextran in control cells and tricellulin knockdown clones. Tricellulin knockdown increased the permeability to 4-kDa FITC-dextran under an osmotic condition (n=7, *** $p \leq 0.001$).

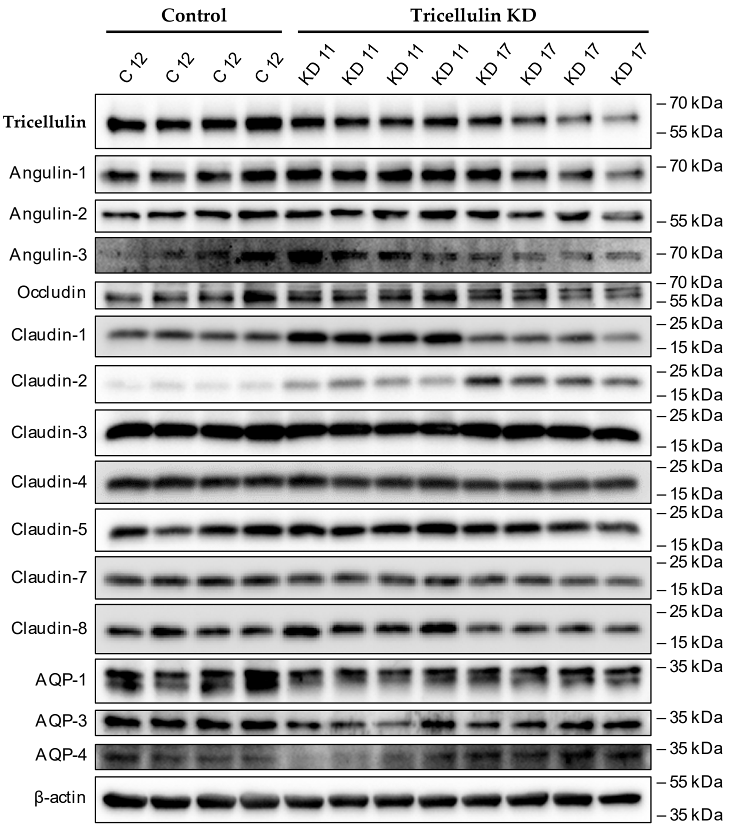

(a)

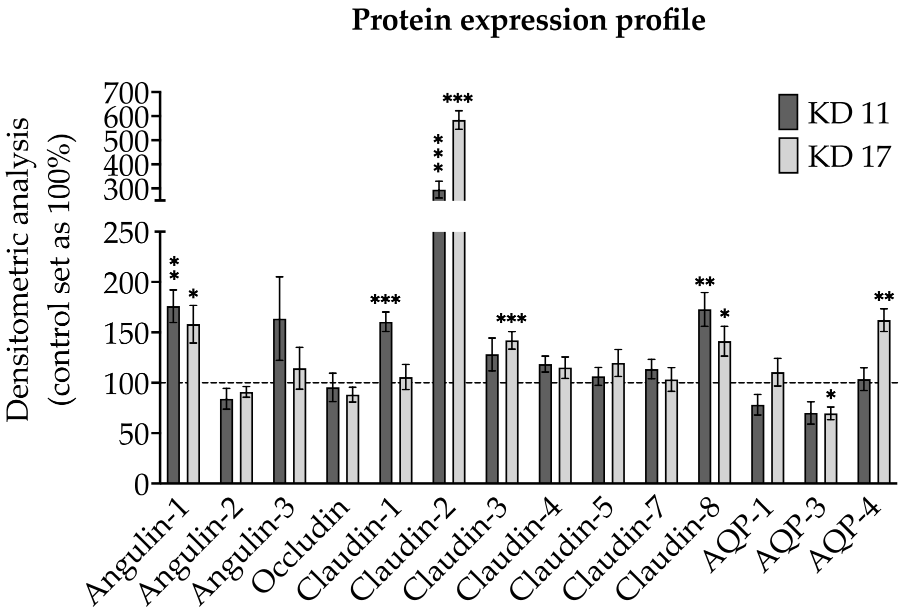

(b)

Figure S3. Angulin, occludin, claudin and AQP expression in control and tricellulin knockdown HT-29/B6 cells. **(a)** Representative Western blots. **(b)** Densitometric analysis of protein expression levels in stable shTRIC transfectants in comparison to the vector-transfected control. β-actin was used as an internal control for normalization to protein content. (n=4-14, N=4, * $p \leq 0.05$, ** $p \leq 0.01$, *** $p \leq 0.001$).

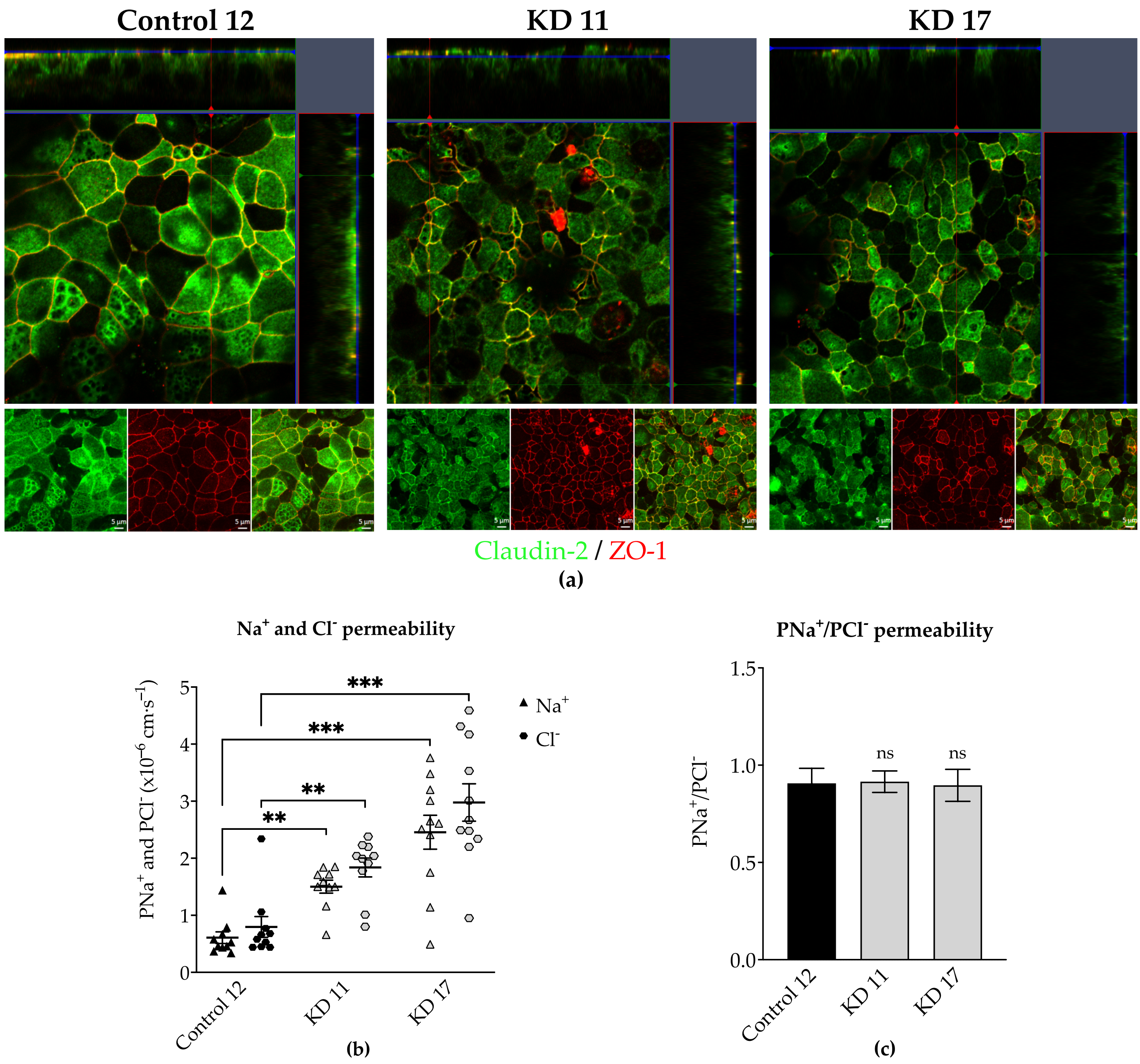

Figure S4. Functional analysis of tricellulin knockdown HT-29/B6 cells. **(a)** Localization of claudin-2 in tricellulin knockdown clones. Claudin-2 localize at the apical membrane, subjunctional membrane and cytoplasm without differences between the knockdown clones and their control (claudin-2: green; ZO-1: red). **(b-c)** Effect of upregulation of claudin-2 on permeability for Na^+ and Cl^- ions. **(b)** Na^+ and Cl^- permeability is increased in both KD clones without any change in selectivity (n=11, ** $p \leq 0.01$, *** $p \leq 0.001$) and **(c)** Ratio Na^+ over Cl^- permeability did not change in KD clones compare with the control (n=11, ns: not significant). The upregulated claudin-2 is non-functional and did not change the cation selectivity of TJ in HT-29/B6 cells.
